# *Legionella pneumophila* CsrA is a pivotal repressor of transmission traits and activator of replication

**DOI:** 10.1046/j.1365-2958.2003.03706.x

**Published:** 2003-10

**Authors:** Ari B. Molofsky, Michele S. Swanson

**Affiliations:** Department of Microbiology and Immunology, University of Michigan Medical School, Ann Arbor, MI 48109–0620, USA

## Abstract

*Legionella pneumophila* can replicate inside amoebae and also alveolar macrophages to cause Legionnaires’ Disease in susceptible hosts. When nutrients become limiting, a stringent-like response coordinates the differentiation of *L. pneumophila* to a transmissive form, a process mediated by the two-component system LetA/S and the sigma factors RpoS and FliA. Here we demonstrate that the broadly conserved RNA binding protein CsrA is a global repressor of *L. pneumophila* transmission phenotypes and an essential activator of intracellular replication. By analysing *csrA* expression and the phenotypes of *csrA* single and double mutants and a strain that expresses *csrA* constitutively, we demonstrate that, during replication in broth, CsrA represses every post-exponential phase phenotype examined, including cell shape shortening, motility, pigmentation, stress resistance, sodium sensitivity, cytotoxicity and efficient macrophage infection. At the transition to the post-exponential phase, LetA/S relieves CsrA repression to induce transmission phenotypes by both FliA-dependent and -independent pathways. For *L. pneumophila* to avoid lysosomal degradation in macrophages, CsrA repression must be relieved by LetA/S before phagocytosis; conversely, before intracellular bacteria can replicate, CsrA repression must be restored. The reciprocal regulation of replication and transmission exemplified by CsrA likely enhances the fitness of microbes faced with fluctuating environments.

## Introduction

To survive, microbes are adept at sensing environmental changes and altering their physiology. Concomitant with alterations in bacterial metabolism, growth rate, and stress resistance, pathogenic microbes must regulate virulence effectors that promote either replication or transmission. A strategy shared by a number of intracellular pathogens is to alternate between replicative, intracellular forms and resilient, infectious extracellular forms that avoid immune-mediated destruction. For example, the obligate intracellular bacterium *Chlamydia trachomatis* differentiates from an intracellular replicative form, RB, to a highly resistant and infectious form, EB. These two forms have distinct morphologies and properties, indicating widespread changes in gene expression (Shaw et al., 2000). By coupling developmental pathways to external cues, microbes can adapt to a variety of environments.

*Legionella pneumophila*, a Gram-negative facultative intracellular pathogen, is commonly found in aquatic environments, where protozoa are its natural hosts (Rowbotham, 1980; Fields, 1996). However, when contaminated water is inhaled by susceptible individuals, *L. pneumophila* can also replicate within human alveolar macrophages and cause a progressive pneumonia called Legionnaires’ Disease (McDade et al., 1977; Horwitz and Silverstein, 1980). Similar to *C. trachomatis*, *L. pneumophila* alternates between an infectious, non-replicating form thought to promote transmission to a new host, and an intracellular replicative form which does not express transmission traits (Rowbotham, 1986; Byrne and Swanson, 1998). In broth cultures, as amino acids become limiting, *L. pneumophila* stops replicating and activates transmission traits, including cytotoxicity, motility, osmotic and heat resistance, sodium sensitivity, and the capability to avoid phagosome-lysosome fusion (Byrne and Swanson, 1998; Hammer and Swanson, 1999). Therefore, we have used exponential (E) and post-exponential (PE) phase broth cultures as a tool to model the replicative and transmissive phases, respectively, of the *L. pneumophila* lifecycle in nature.

As *L. pneumophila* coordinately regulates motility with other virulence-associated traits, by screening for strains defective for expression of a flagellin reporter plasmid, mutants were identified that are also defective for other transmission traits. By this approach, the two-component system LetA/S and the sigma factor FliA were demonstrated to cooperate with the stationary phase sigma factor RpoS to coordinate differentiation of replicative *L. pneumophila* to a transmissive form (Bachman and Swanson, 2001; Hammer et al., 2002). According to our strin-2003 Blackwell Publishing Ltd gent response model of *L. pneumophila* growth phase-dependent differentiation (Byrne and Swanson, 1998; Bachman and Swanson, 2001; Hammer et al., 2002), as the microbes deplete nutrients from their eukaryotic host cell, the ribosomal enzyme RelA senses low bacterial amino acid levels through elevated amounts of uncharged tRNAs and synthesizes the alarmone ppGpp. In turn, ppGpp activates the sigma factor RpoS and the two-component system LetA/S to induce subsets of transmission genes (Bachman and Swanson, 2001; Hammer et al., 2002). RpoS and LetA/S cooperate to regulate certain virulence-associated traits, such as motility and sodium sensitivity, but each regulator also acts independently to control other aspects of transmission.

In *Pseudomonads* and *Erwinia caratovora*, the LetA/S homologues GacA/S and ExpA/S activate synthesis of exported virulence effectors by counteracting the global regulatory protein CsrA (Blumer et al., 1999; Aarons et al., 2000; Cui et al., 2001; Heeb and Haas, 2001; Heeb et al., 2002). In particular, these two-component systems induce expression of CsrB homologues, which are non-coding regulatory RNAs that can sequester multiple copies of CsrA protein, thereby inducing CsrA-repressed virulence effectors. In general, the CsrA protein functions as a modulator of mRNA stability and translation, whereas the CsrB RNA binds and sequesters multiple copies of CsrA protein to de-repress CsrA targets.

Originally described as an *Escherichia coli* regulator of glycogen accumulation (Romeo et al., 1993), the small RNA-binding protein Carbon Storage Regulator A (CsrA) and the non-coding repressor RNA CsrB are now known to function as a global post-transcriptional regulatory system in a large number of bacterial species (reviewed in Romeo, 1998). CsrA regulates a host of *E. coli* PE phase traits, including central carbohydrate metabolism, motility, biofilm formation, and adherence (Sabnis et al., 1995; Yang et al., 1996; Wei et al., 2000; 2001; Jackson et al., 2002). For the *E. coli* glycogen biosynthesis gene *glgC*, CsrA binds near the ribosomal binding site of the mRNA, preventing ribosomal binding and destabilizing the transcript (Baker et al., 2002). CsrA can also stabilize RNA transcripts, such as the *E. coli* master flagellar regulator *flhDC*, but the mechanism for this stabilization is not thoroughly understood (Wei et al., 2001). In the plant pathogen *Erwinia carotovora*, the CsrA homologue RsmA affects production of several lytic enzymes, motility and quorum sensing, whereas CsrA of *Salmonella typhimurium* regulates the Salmonella pathogenecity island 1, including genes important for cell invasion (Chatterjee et al., 1995; Cui et al., 1995; Altier et al., 2000a,b). *Pseudomonas aeruginosa* RsmA represses extracellular virulence effectors and production of quorum sensing genes during exponential growth (Pessi et al., 2001). In *L. pneumophila*, constitutive expression of wild-type *csrA* inhibits the PE phase traits motility and pigment production (Fettes et al., 2001). In sum, the CsrA/B global regulatory system is widely conserved and functions to repress PE phase virulence traits in many pathogenic bacteria.

In this work, we extend our stringent response model for *L. pneumophila* differentiation by proposing that CsrA functions as a pivotal regulator of the *L. pneumophila* lifecycle. By analogy to homologous regulatory circuits, we postulated that during the replicative phase CsrA represses transmission traits and coordinately promotes replication. When *L. pneumophila* sense that amino acid levels are low, activated LetA/S would relieve CsrA repression of PE traits, including several required for virulence. To test this model, we constructed both *csrA* constitutive expression and *csrA* conditional mutant strains in wild type and a panel of regulatory mutant backgrounds and analysed their expression of transmissive phase traits.

## Results

### *Identification of* csrA

Fettes et al. (2001) recently identified the *L. pneumophila csrA* gene and demonstrated that, in high copy, the wild-type locus inhibits expression of the PE phase traits motility, pigment production and cell shortening. To extend this analysis by examining its regulation of *L. pneumophila* differentiation and virulence, we first cloned *L. pneumophila csrA* and verified its ability to complement the glycogen storage phenotype of *E. coli csrA* mutants as described previously (data not shown, Fettes et al., 2001).

### Legionella pneumophila *express* csrA *during the replication phase*

**Fig. 1.**
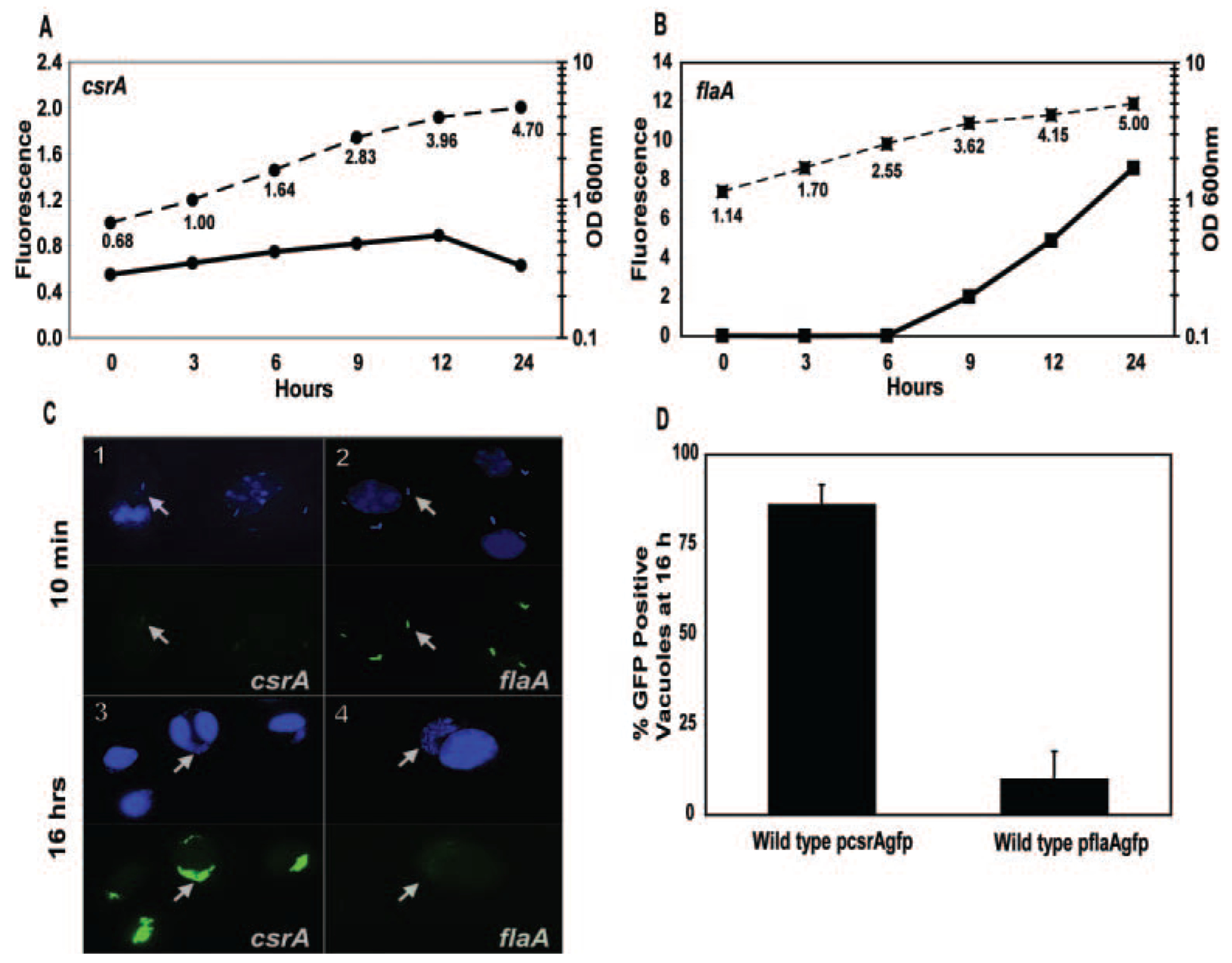
Reciprocal expression of *csrA* and *flaA*. A. *csrA* is expressed by *L. pneumophila* replicating in broth. To monitor *csrA* promoter activity, wild-type Lp02 p*csrA*gfp (MB469) was cultured in broth, then at the times indicated bacterial density was quantified by measuring OD_600nm_ by spectrophotometry (dashed lines) and Green Fluorescence Protein (GFP) accumulation by fluorometry (solid lines). Shown is a growth curve beginning at mid-exponential phase that is representative of multiple cultures in two independent experiments. B. *flaA* is expressed by PE phase broth cultures of *L. pneumophila*. To monitor promoter activity of *flaA*, an established marker of the transmissive phase, wild-type Lp02 p*flaA*gfp (MB355) was analysed as described in A. Note that the Y-axes of panels A and B are of different scale, as the GFP expression of *flaAgfp* is eight- to tenfold higher than that of *csrAgfp*. Shown is a growth curve beginning at mid-exponential phase that is representative of multiple cultures in two independent experiments. The fluorescence of a promoterless *gfp* control strain was subtracted from all values shown in A and B. C. Murine bone marrow-derived macrophages were infected with wild-type *L. pneumophila* containing p*csrA*gfp (MB469; left panels) or p*flaA*gfp (MB344; right panels) for 10 min (panels 1 and 2) or 16 h (panels 3 and 4), fixed, then macrophage and bacterial DNA was stained with DAPI (blue; upper panels) and *csrA* or *flaA* promoter activity was visualized by GFP fluorescence (green; lower panels). D. At 16 h after infection, replicating bacteria carrying *csrAgfp* are fluorescent in significantly higher numbers than are replicating cells carrying *flaAgfp* (*P* = 1.6e–6, students’ two-tailed *t*-test). Replication vacuoles, similar to those depicted in C panels 3 and 4, were scored as positive if they contained five or more closely associated *L. pneumophila* of which >50% exhibited fluorescence above background levels. Shown are the mean values ± SD determined from three independent experiments performed in duplicate in each of which 50 or more replicating vacuoles were scored.

Because in other bacteria CsrA inhibits post-exponential (PE) phase genes, including virulence factors, we predicted *L. pneumophila csrA* would be active during the replication period to repress virulence-associated phenotypes. To monitor *csrA* expression during the *L. pneumophila* lifecycle, broth cultures of wild-type microbes transformed with a plasmid containing a *csrA::gfp* transcriptional fusion (WT p*csrAgfp*) were analysed by fluorometry (Fig. 1A). The *csrA* promoter was active throughout the exponential (E) phase, then its activity declined slightly as the microbes progressed into the PE phase. In comparison, expression of the known PE phase flagellar subunit gene *flaA* (Heuner et al., 1995; 1999; Hammer and Swanson, 1999) was undetectable during exponential growth, but was strongly activated as the microbes entered PE phase, and green fluorescent protein (GFP) continued to accumulate (Fig. 1B). Although the stability of GFP precludes strict interpretations of promoter activity, maintenance of fluorescence by replicating bacteria requires constant p*csrAgfp* expression. Therefore, the *csrA* promoter is active preferentially during the bacterial replication period *in vitro*, a conclusion consistent with northern analysis monitoring *csrA* transcript levels during broth growth (Fettes et al., 2001).

To extend the results obtained from our *in vitro* model, *L. pneumophila* expression of p*csrAgfp* and p*flaAgfp* was next compared during its lifecycle in macrophages. Ten minutes after infection with PE phase bacteria, the p*flaAgfp* microbes were strongly fluorescent, whereas the p*csrAgfp* bacteria were only faintly so (Fig. 1C), consistent with the broth culture fluorometry data (Fig. 1A and B). By 16 h after infection, intracellular replicating *L. pneumophila* containing p*csrAgfp* were bright green (Fig. 1C) and most replication vacuoles (86% ± 5.7%) contained a majority of GFP-positive microbes (Fig. 1D). At the same time, replicating p*flaAgfp* microbes displayed little fluorescence (Fig. 1C), and few vacuoles (9.8% ± 7.9%) contained a majority of GFP-positive bacteria (Fig. 1D). The reciprocal pattern of expression observed for *csrA* and the *flaA* transmission phase gene during the *L. pneumophila* lifecycle is consistent with the observation that constitutive *csrA* expression decreases mRNA levels of the flagellar sigma factor *fliA* (Fettes et al., 2001) and with the hypothesis that CsrA is a vital regulator during the replicative phase.

### *Growth and morphology of* L. pneumophila *that either lack or constitutively express* csrA

To test the prediction that CsrA is a replication phase repressor of PE traits, we created strains that lack or constitutively express *csrA*, then determined their transmission phenotypes in both E and PE phases. We and others were unable to recover *csrA* null mutant strains using standard procedures (see Experimental procedures, Fettes et al., 2001), suggesting that *L. pneumophila* requires *csrA* to grow on bacteriological agar. Instead, we created *csrA* conditional null mutants in which the chromosomal *csrA* locus was deleted and the expression of a plasmid-encoded wild-type *csrA* could be induced with IPTG (p206-csrA), hereafter referred to as *csrA* mutants (MB464, MB465). As expected, in the absence of IPTG, *csrA* mutants formed colonies poorly. When cultured in broth that contained IPTG to induce *csrA*, the bacteria replicated at a wild-type rate (Supplementary material, Fig. S1). However, when transferred to broth without IPTG, the yield of *csrA* mutants increased slowly, and the cells were short and coccoid-shaped, resembling PE phase wild-type microbes (Supplementary material, Figs S1 and S2). Furthermore, unlike wild-type Lp02 *L pneumophila*, which become motile at culture densities of OD_600nm_ > 3.0 (Byrne and Swanson, 1998; Hammer and Swanson, 1999), some *csrA* mutant cells swam at densities as low as 0.1; by an OD_600nm_ of 2.0, a majority of *csrA* mutant cells were motile (Table 1).

As a complementary approach to study CsrA function, a wild-type strain in which the *csrA* gene could be constitutively expressed with addition of IPTG (wild-type pcsrA) was constructed and examined for morphology and motility. Unlike p206-csrA, used in the conditional *csrA* mutants, pcsrA has an IPTG-responsive P_tac_ promoter that drives high-level expression of *csrA*, and was therefore appropriate for studies of constitutive *csrA* expression. Even in the absence of IPTG induction, the pcsrA microbes displayed a leaky *csrA* expression, based on their elongated cell shapes and partially repressed transmission phase phenotypes, including motility, infectivity, sodium-sensitivity, and cytotoxicity (data not shown). All subsequent experiments with constitutive *csrA* expressing microbes (wild-type pcsrA) maintained the cultures with IPTG throughout broth growth. When induced with IPTG, *L. pneumophila* wild-type pcsrA cells grew as well as wild-type bacteria in broth (data not shown). However, when compared to wild-type *L. pneumophila*, the cells constitutively expressing *csrA* due to IPTG induction became even more elongated, similar to bacteria lacking the two-component response regulator *letA*, and their motility was reduced and *flaA* expression was <30% of wild-type levels (Table 1, Fig. S2, Fettes et al., 2001). Observations of cells that lack or that constitutively express *csrA* are consistent with a model whereby *L. pneumophila* produce CsrA to repress a host of PE traits during the replicative period; during the transmissive phase, CsrA repression must be relieved to activate motility and cell shortening.

### *CsrA inhibits pigment production and stress resistance of PE phase* L. pneumophila

As *L. pneumophila* progresses through the PE phase, it produces a melanin-like, soluble pigment that protects these aquatic microbes from UV damage but is not required for intracellular growth (Warren and Miller, 1979; Wiater et al., 1994; Wintermeyer et al., 1994; Steinert et al., 1995). Our model predicts that when RelA senses amino acid starvation, it produces ppGpp to activate the two-component system LetA/S, which in turn represses CsrA activity to induce expression of PE phase traits. *Legionella pneumophila* that lack *relA* or constitutively express *csrA* are defective for pigment accumulation (Fettes et al., 2001; Zusman et al., 2002). Therefore, we predicted that *letA* and *letS* mutants produce little pigment.

Wild-type *L. pneumophila* and *rpoS* mutants accumulated substantial pigment, as demonstrated previously (Supplementary material, Fig. S3A; Table 1; Hales and Shuman, 1999; Bachman and Swanson, 2001). As predicted, *letA* or *letS* mutants accumulated little pigment, a phenotype similar to that observed for the constitutively active *csrA* strain (Supplementary material, Fig. S3B; Table 1, Fettes et al., 2001). In addition, compared to wild-type microbes, bacteria that either lacked the LetA or LetS activators or constitutively expressed the CsrA repressor exhibited a significant loss of absorbance at OD_600nm_ as cultures progressed into late PE, yet a corresponding loss in CFU was not observed during the same period (data not shown). More detailed studies are required to determine whether the decline in OD_600nm_ is due to further changes in bacterial shape or small amounts of cell lysis difficult to detect by CFU assays.

As *L. pneumophila* alter their physiology to accommodate nutrient limitation, resistance to various environmental stresses is activated (Bandyopadhyay and Steinman, 1998; Hales and Shuman, 1999; Hammer and Swanson, 1999; Bachman and Swanson, 2001). *Legionella pneumophila letA* mutants are deficient in stress resistance, as are *E. coli* that lack the LetA orthologue UvrY (Pernestig et al., 2001; Lynch et al., 2003). As LetA activates and CsrA represses a number of PE phase traits of *L. pneumophila*, we tested the prediction that CsrA also represses resistance to environmental stress.

Wild-type PE phase *L. pneumophila* tolerated both heat and osmotic stress well, whereas E phase microbes were sensitive (Table 1, Hales and Shuman, 1999; Hammer and Swanson, 1999). As predicted, bacteria that constitutively expressed *csrA* remained sensitive to heat and osmotic stress in the PE phase, resembling both E phase wild-type microbes and PE phase *letA* mutants. Conversely, when E phase *csrA* mutants were subjected to heat, they were partially heat resistant; as they entered the PE phase, the *csrA* mutants became fully heat resistant (Table 1). Thus, *csrA* represses heat resistance during replication, but other factors may also activate heat resistance of PE phase *L. pneumophila*. When subjected to pH or oxidative stress, both *letA* mutants and *csrA* constitutive expressing microbes survived as efficiently as PE phase wild type and *rpoS* mutant cultures, whereas E phase wild-type microbes were sensitive (data not shown, Hales and Shuman, 1999; Bachman and Swanson, 2001). However, Lynch et al. (2003) report that *letA* mutants are sensitive to pH and oxidative stress; the most likely explanation for this discrepancy is strain differences. In sum, CsrA represses several physiological changes characteristic of the PE phase, but other factors may also regulate some PE phase traits, including tolerance of heat, acid and oxidative stress (Table 1). Alternately, it is possible that our *csrA* conditional null mutant retains low levels of IPTG-independent *csrA* expression, and these traits may be repressed by low levels of CsrA that do not repress the majority of transmission traits.

### *CsrA represses cytotoxicity and sodium-sensitivity, two* L. pneumophila *virulence traits*

**Fig. 2.**
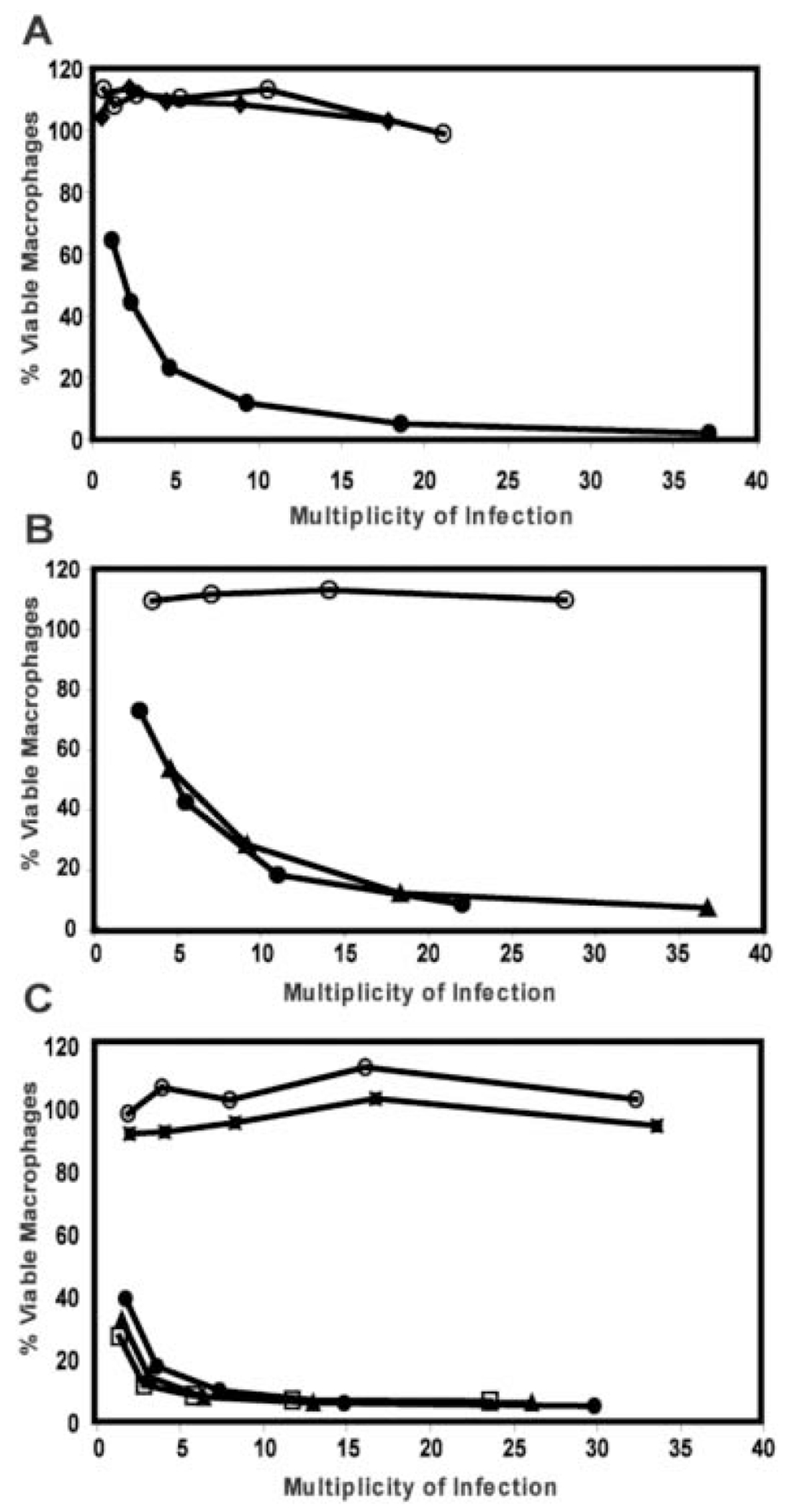
CsrA represses *L. pneumophila* contact-dependent cytotoxicity. A. Bacteria were incubated with macrophages at the multiplicity of infection shown for 1 h, then macrophage viability was quantified by the reduction of the colorimetric dye Alamar Blue. Wild-type Lp02 (MB473) cultured to the PE phase (solid circles) was cytotoxic, but wild-type Lp02 pcsrA cultured with IPTG to the PE phase (MB472; solid diamonds) resembled the non-cytotoxic control, E phase wild-type Lp02 (MB473; open circles). B. E phase *csrA::kan* mutant microbes are cytotoxic (MB464, solid triangles), resembling PE phase wild-type microbes (MB463, solid circles), and the negative control E phase wild-type strain is not (MB463, open circles). C. Both PE phase *csrA::kan* mutant microbes (MB464, solid triangles) and PE phase *csrA letA* double mutants (MB466, open squares) are cytotoxic, but PE phase *letA* single mutants are not (MB413, solid squares). PE and E phase wild type controls are labelled as in A and B. Shown are representative graphs from three or more independent experiments performed in duplicate or triplicate.

As *L. pneumophila* coordinates expression of virulence with general PE phase traits, we postulated that CsrA also represses virulence-associated traits during the replication period. In the PE phase, wild-type *L. pneumophila* are cytotoxic to bone marrow-derived macrophages, whereas E phase bacteria are not (Byrne and Swanson, 1998). This contact-dependent cytotoxicity depends upon the Dot/Icm type IV secretion system and the PE phase activator proteins LetA, LetS, and FliA (Kirby et al., 1998; Hammer et al., 2002) and may aid in bacterial escape from spent hosts (Alli et al., 2000). Consistent with our model, wild-type PE phase cultures were highly cytotoxic, whereas PE phase cells that constitutively express *csrA* were not, comparable to E phase wild-type bacteria (Fig. 2A). Conversely, *csrA* mutants in the E phase were prematurely cytotoxic, comparable to PE phase wild type (Fig. 2B).

**Fig. 3.**
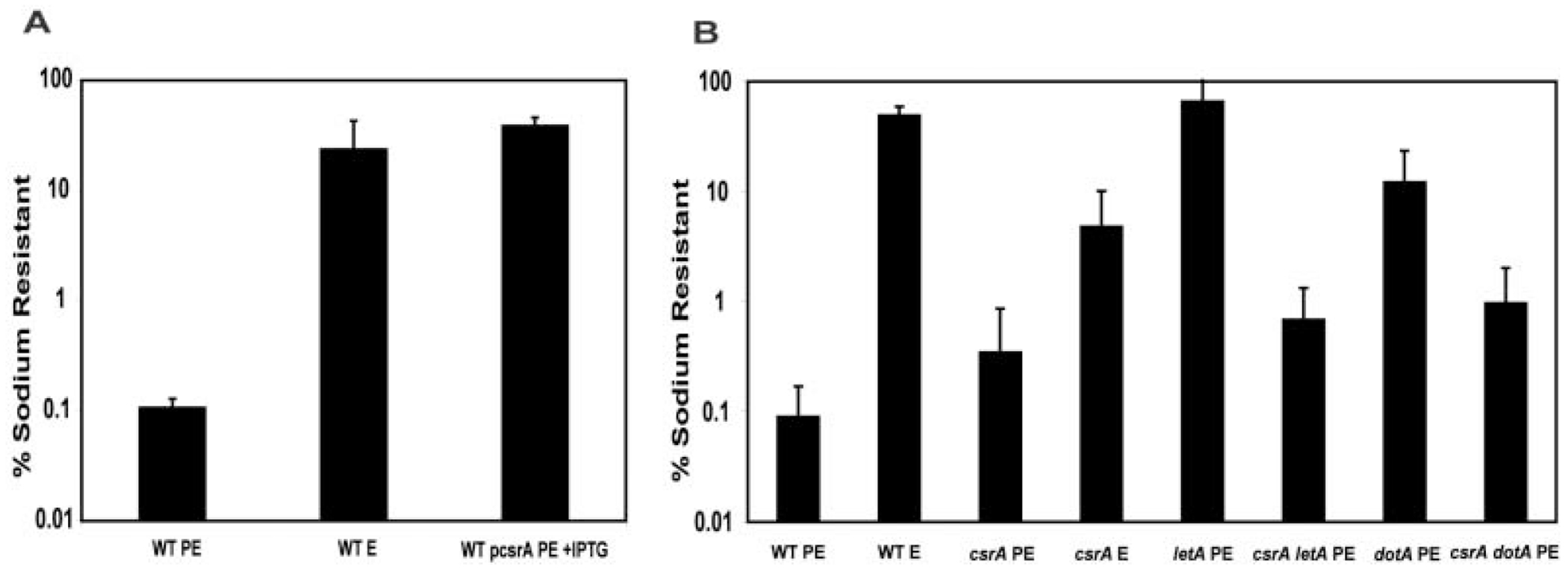
Sodium sensitivity is repressed by CsrA and activated by LetA. A. PE phase wild-type Lp02 (MB473) are sensitive to NaCl, but Lp02 pcsrA cultured with IPTG to the PE phase (MB472) are not, comparable to the E phase wild-type control (MB473). B. *csrA::kan* mutants (MB464) are partially sodium-sensitive during E phase, becoming fully sensitive in the PE phase, similar to PE wild-type Lp02 (MB463). PE phase *letA* (MB413) and *dotA* (MB460) mutants are sodium resistant, whereas *csrA letA* (MB466) and *csrA dotA* (MB468) double mutants are sodium sensitive. Sodium resistance was quantified for E or PE phase cultures by plating on medium with or without 100 mM NaCl, then calculating [(CFU on CYET + 100 mM NaCl)/(CFU on CYET)] × 100. Shown are the average means ± SD calculated from two to four independent experiments.

Virulent *L. pneumophila* are sensitive to sodium, as judged by their poor plating efficiency on agar containing 100 mM NaCl (Catrenich and Johnson, 1989). Although the mechanism is not understood, several of the *dot/icm* type IV secretion mutants were originally identified on the basis of their growth in high NaCl concentrations, and LetA/S activates sensitivity to sodium in the PE phase (Sadosky et al., 1993; Vogel et al., 1996; Byrne and Swanson, 1998; Hammer et al., 2002). As predicted, PE phase cells that constitutively express *csrA* remained resistant to sodium (Fig. 3A), comparable to E phase wildtype control cultures and PE phase *letA* mutants (Fig. 3B, Hammer et al., 2002). Conversely, E phase *csrA* mutants were partially sodium sensitive, becoming fully sodium sensitive in the PE phase (Fig. 3B). Therefore, the virulence-associated traits of cytotoxicity and sodium-sensitivity are under CsrA-mediated repression during replication; upon transition into the PE phase, this repression must be relieved to allow expression of virulence traits.

### LetA induces PE phase transmission traits by relieving CsrA repression

By analogy to homologous regulatory circuits in other Gram-negative bacteria, CsrA repression of *L. pneumophila* transmission traits is predicted to be relieved in the PE phase by activation of the two-component system LetA/S. If so, genetic inactivation of CsrA should bypass the requirement for LetA/S in transmission phenotype expression. To test this aspect of our model, we mutated the *letA* locus in the *csrA* conditional null background, then analysed the phenotype of *csrA letA* double mutants by culturing the cells in broth that lacked IPTG.

**Fig. 4.**
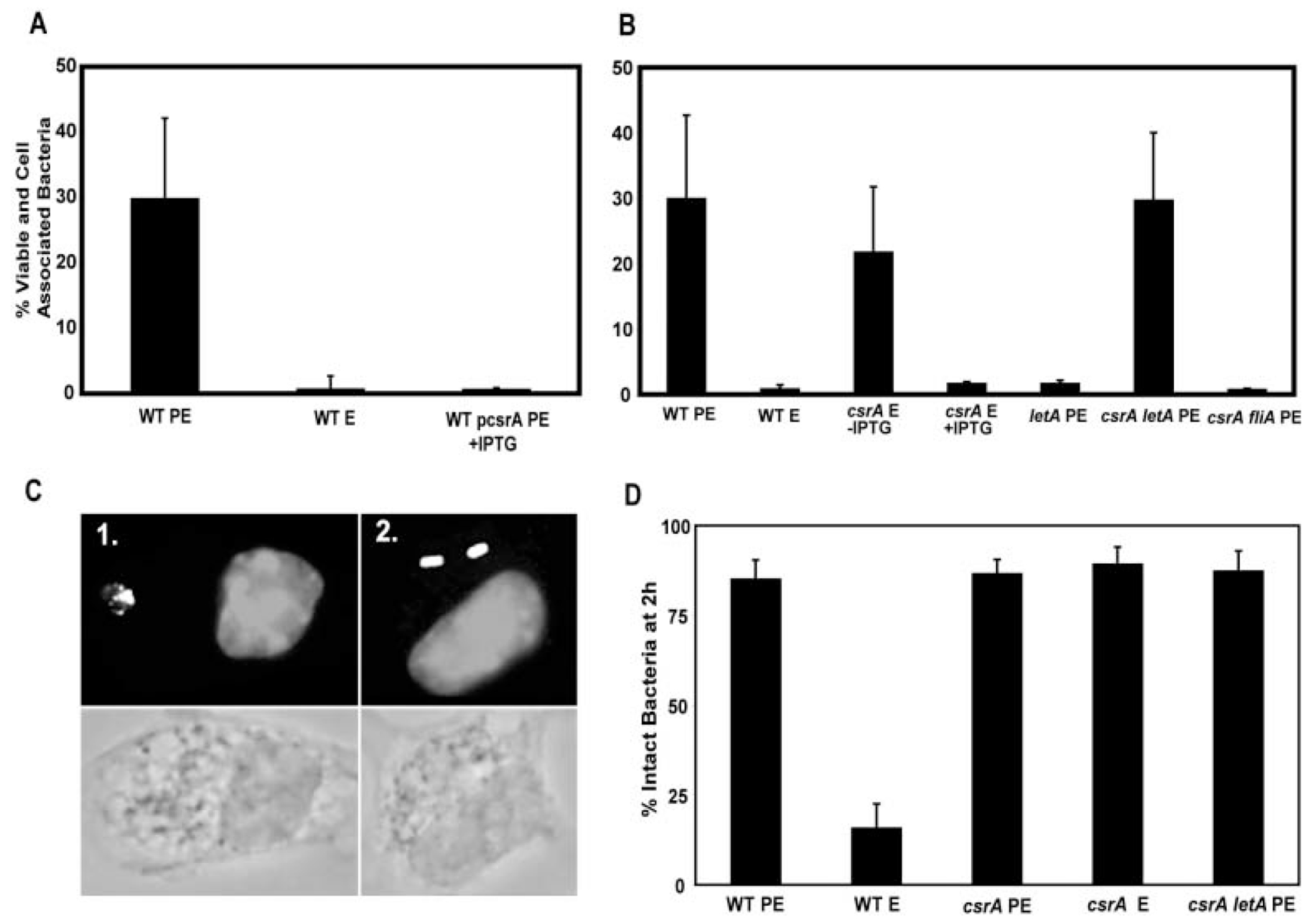
CsrA repression of *L. pneumophila* infectivity is relieved by LetA. A and B. The ability of bacteria to bind, enter and survive in macrophages was quantified as [(CFU of monolayer at 2 h CFU^−1^ of inoculum at 0 h)] × 100. PE phase wild-type Lp02 were highly infectious (MB473 in A, MB463 in B), whereas E phase wild-type was not. PE phase Lp02 pcsrA (MB472, A) and E phase *csrA* conditional mutants cultured with IPTG (MB464, B) were not infectious, nor was PE phase letA (MB413, B) or *csrA fliA* double mutants (MB467, B). E phase *csrA* mutants cultured without IPTG (MB464, B) and PE phase *csrA letA* double mutants (MB466, B) were infectious to macrophages. C. To corroborate results of the CFU assay (A and B), the percent of intact bacteria at 2 h was quantified by fluorescence microscopy. Shown are representative images of degraded E phase wild-type microbes (panel 1) and intact E phase *csrA::kan* mutants (panel 2) double-labeled with the DNA stain DAPI to visualize macrophage nuclei and intact bacteria and by immunofluorescence to visualize intact and degraded *L. pneumophila* (upper panels) and the corresponding brightfield images (lower panels). D. The per cent of intracellular bacteria that were intact was quantified for samples prepared as in C, with E and PE phase wild-type microbes serving as controls. The average means ± SD calculated from three to four independent experiments are shown (A, B and D).

When CsrA repression was relieved by mutation, the LetA activator was dispensable for *L. pneumophila* to express every transmission phenotype examined. After reaching the PE phase, both the *csrA* single mutant and the *csrA letA* double mutant became coccoid, fully motile, and pigmented, comparable to wild-type *L. pneumophila* (Table 1). Loss of *csrA* also restored to PE phase *letA* mutants heat resistance (Table 1), cytotoxicity (Fig. 2C), and sodium-sensitivity (Fig. 3B). Finally, the *csrA letA* double mutants were as infectious for macrophages as PE phase wild-type *L. pneumophila* (Fig. 4B), as they efficiently avoided lysosomal degradation (Fig. 4D), traits discussed in detail below. The observation that loss of CsrA activity bypassed all of the *letA* mutant transmission defects indicates that, for the phenotypes assayed, LetA solely functions to repress CsrA activity, thereby inducing transmission traits.

As a test for specificity of the genetic suppression observed, we asked whether loss of the CsrA repressor also compensated for another pleiotropic mutation, *dotA*. As a putative integral component of the type IV secretion complex, DotA is thought to be required for delivery of virulence factors to the host cell (Berger et al., 1994; Roy and Isberg, 1997; Roy et al., 1998; Vogel et al., 1998). Accordingly, loss of the CsrA repressor should not bypass the virulence defects of *dotA* secretion mutants. As predicted, loss of *csrA* did not restore cytotoxicity to *dotA* mutant microbes: After 1 h at an MOI of 25, only ~5% of macrophages incubated with PE wild-type microbes were viable, whereas ~95% of macrophages incubated with PE *dotA* single mutants or *csrA dotA* double mutants were viable. However, PE phase *csrA dotA* double mutants were 10-fold more sodium-sensitive than PE phase *dotA* single mutants (Fig. 3B), indicating either that loss of *csrA* non-specifically causes partial restoration of sodium-sensitivity or that a genetic link exists between *csrA* and *dotA* that remains to be understood. Both PE phase *dotA* single mutants and *csrA dotA* double mutants were resistant to heat and secreted the melanin-like pigment (Table 1), demonstrating that the type IV secretion apparatus is not required for stress resistance or pigment production. Even in the absence of a functional type IV secretion system, *L. pneumophila* required *csrA* to repress motility and to replicate at wildtype rates (data not shown), suggesting that CsrA may also activate the replication phenotype.

### CsrA repression is mediated by both FliA-dependent and -independent pathways

CsrA is postulated to repress the flagellar sigma factor FliA, thereby inhibiting motility and certain virulence traits, based on the loss of *fliA* mRNA when *csrA* is expressed constitutively (Fettes et al., 2001). To test genetically whether other CsrA-repressed transmission traits are dependent upon FliA activation, we constructed and analysed *csrA fliA* double mutants in a *csrA* conditional null background.

As observed for the *csrA*, *csrA dotA* and *csrA letA* mutant strains, the *csrA fliA* double mutant strain also had an apparent slow growth rate, illustrating that CsrA function is critical for *L. pneumophila* replication in broth (data not shown). However, when it eventually reached high culture densities, the *csrA fliA* double mutant strain was not motile, infectious for macrophages, or cytotoxic (Table 1; Fig. 4B; data not shown). Therefore, even when CsrA repression is relieved by mutation, *L. pneumophila* requires the flagellar sigma factor FliA to express three of its transmission traits.

Other transmission traits are expressed by *L. pneumophila* independently of FliA. In the PE phase, both *fliA* single and *csrA fliA* double mutants were coccoid-shaped and sodium sensitive (Hammer et al., 2002; data not shown). Likewise, when subjected to a variety of environmental stresses, both the *fliA* and *csrA fliA* mutants resembled PE phase wild-type and *rpoS* cultures, unless they constitutively expressed *csrA* (Table 1; Hales and Shuman, 1999; Bachman and Swanson, 2001). Therefore, neither the FliA nor the RpoS sigma factors are required for the general resilience of PE phase *L. pneumophila*.

Unexpected effects of FliA and LetA activity on pigment production were observed. When compared to PE phase wild-type *L. pneumophila*, *fliA* mutants were hyper-pigmented (data not shown), suggesting FliA either directly or indirectly represses pigment production. Although constitutive expression of *csrA* by wild type, *rpoS*, or *fliA* mutants repressed pigment production, hyper-accumulation of pigment was observed when *letA* mutants were induced to express *csrA* constitutively (Table 1), even though this strain is predicted to have high levels of CsrA activity unopposed by LetA-dependent inhibition. Additional experiments are required to understand the excess pigmentation in supernatants of strains that lack FliA or have deregulated CsrA activity.

### CsrA is necessary for intracellular growth, but dispensable for initial infection

Studies of broth cultures indicate that during the replication period, CsrA represses a range of PE traits, including those likely to promote transmission to a new host. To assess the validity of our interpretations generated from *in vitro* studies, macrophages were infected with *L. pneumophila csrA* mutant or constitutively expressing microbes, then the efficiency of infection and intracellular replication were quantified.

When ingested by macrophages, many bacteria and inert particles are swiftly delivered to lysosomal compartments and degraded. However, as *L. pneumophila* sense amino acid starvation and transit into the PE state, they become competent to evade delivery to bactericidal lysosomes for several hours (Horwitz, 1983; Byrne and Swanson, 1998; Sturgill-Koszycki and Swanson, 2000). Unlike highly infectious PE phase *L. pneumophila* (30% ± 12%), PE phase bacteria that constitutively express *csrA* exhibited low infectivity (0.56% ± 0.31%), similar to the E phase wild-type cultures (Fig. 4A). In contrast, in the E phase, *csrA* mutants were highly infectious (22% ± 10%), similar to wild-type PE phase microbes (Fig. 4B). To verify that efficient macrophage infectivity reflects their capacity to evade lysosomal degradation, we assayed by fluorescence microscopy the fate of *L. pneumophila* 2 h after infection of macrophages. As expected, <20% of E phase wild-type bacteria avoided degradation (Fig. 4C and D) and >80% of PE phase wild-type bacteria remained intact (Fig. 4D). In contrast, whether cultured to either the E or PE phase, >80% of *csrA* mutants retained their rod shape 2 h after ingestion (Fig. 4C and D). Thus, in the absence of CsrA activity, *L. pneumophila* become highly infectious and avoid immediate lysosomal degradation, as judged by both the CFU-dependent infectivity assays and direct microscopic inspection (Fig. 4).

**Fig. 5.**
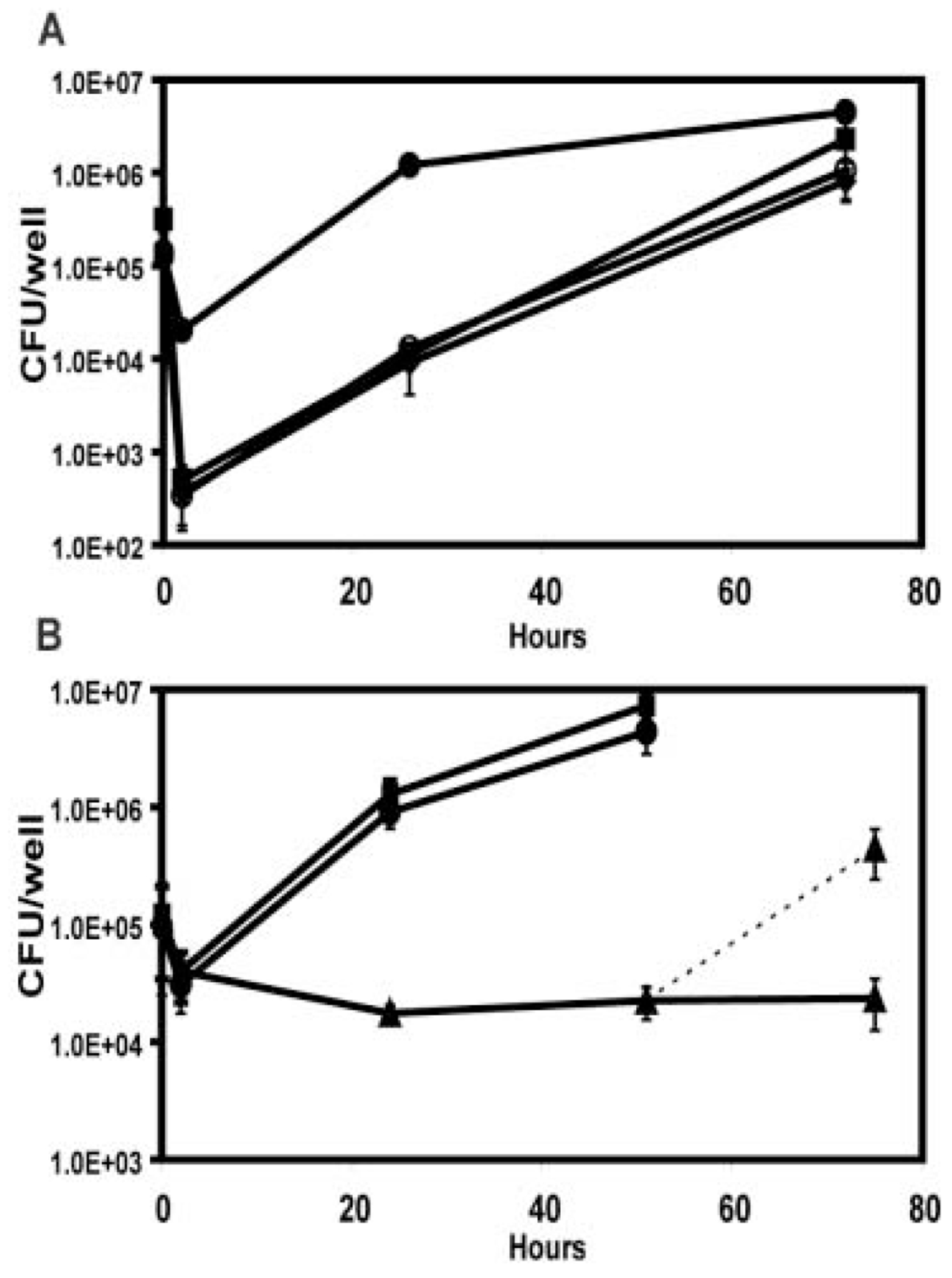
CsrA is essential for *L. pneumophila* growth in macrophages, but its repression must be relieved for efficient transmission. A. Constitutive *csrA* expression, due either to induction of pcsrA or deletion of *letA*, inhibits initial infection of macrophages but not intracellular growth. Macrophages were infected for 2 h at an MOI of ~1.0 with either wild-type Lp02 (MB473) cultured to the E (hollow circles) or PE phase (solid circles), wild-type Lp02 transformed with *pcsrA* and cultured with IPTG to PE phase (MB472; diamonds), or PE mutant *letA* carrying the vector (MB434, squares). At each time indicated, the total CFU per well was quantified. Shown are the mean CFU ± SD calculated from duplicate wells in one of three independent experiments. B. *csrA* mutants cannot replicate in macrophages until *csrA* expression is restored. CFU were quantified as described in A after infection of macrophages with the PE phase wild-type control strain (MB463, circles) or E phase *csrA::kan* mutants untreated (MB464; triangles, solid line) or induced with IPTG either at the time of macrophage infection (squares) or 48 h post infection (triangles; dashed line). Shown are the mean bacterial CFU ± SD determined at the times indicated for duplicate wells in one experiment; similar results were obtained in three to four independent experiments.

Although PE phase *L. pneumophila* that constitutively express *csrA* are poorly infectious (Figs 4A and 5A), those bacteria that survived the initial infection then multiplied at wild-type rates, as judged by the similar slopes of the respective growth curves from 2 to 24 h (Fig. 5A). As predicted, *letA* mutants behaved identically to *csrA* constitutively expressing cells in assays of infectivity and intracellular growth (Figs 4B and 5A; Hammer et al., 2002). Therefore, either constitutive *csrA* production or deletion of *letA* prevented expression of PE phase traits necessary for efficient infection, but neither inhibited intracellular growth, consistent with the proposed role for CsrA as an essential repressor of transmissive phase traits and activator of replication. As the phenotypic patterns observed are identical to E phase wild-type bacteria, we postulate that excess CsrA, either from loss of LetA or induction of pcsrA, phase-locks *L. pneumophila* in the replicative form.

The reciprocal phenotype was observed when macrophages were infected with *L. pneumophila* that lack *csrA*. Macrophages were incubated with E phase *csrA* mutants cultured in broth without IPTG. After 2 h, the percentage of viable and cell-associated *csrA* mutant bacteria was comparable to that observed in parallel infections with the virulent control, wild-type PE phase cultures (Fig. 4B). Thus, unlike E phase wild-type *L. pneumophila*, E phase *csrA* mutants are highly infectious, consistent with the premature expression of genes necessary for *L. pneumophila* to efficiently enter macrophages and delay delivery to phagolysosomes. Nevertheless, in the absence of *csrA*, the highly infectious *L. pneumophila* failed to replicate, even when incubated in macrophages for 72 h (Fig. 5B). In contrast, when IPTG was supplied at the time of macrophage infection, E phase *csrA* mutant bacteria mimicked wild-type PE phase cultures by infecting and also replicating efficiently (Fig. 5B). If instead E phase *csrA* mutant cells were incubated with IPTG both in broth culture and during macrophage infection, they behaved like wild-type E phase cultures (data not shown). We conclude that initially *L. pneumophila* must repress CsrA activity to infect macrophages efficiently; subsequently, CsrA activity must be induced to promote efficient replication.

### csrA *mutants are viable 48 h post infection*

**Fig. 6.**
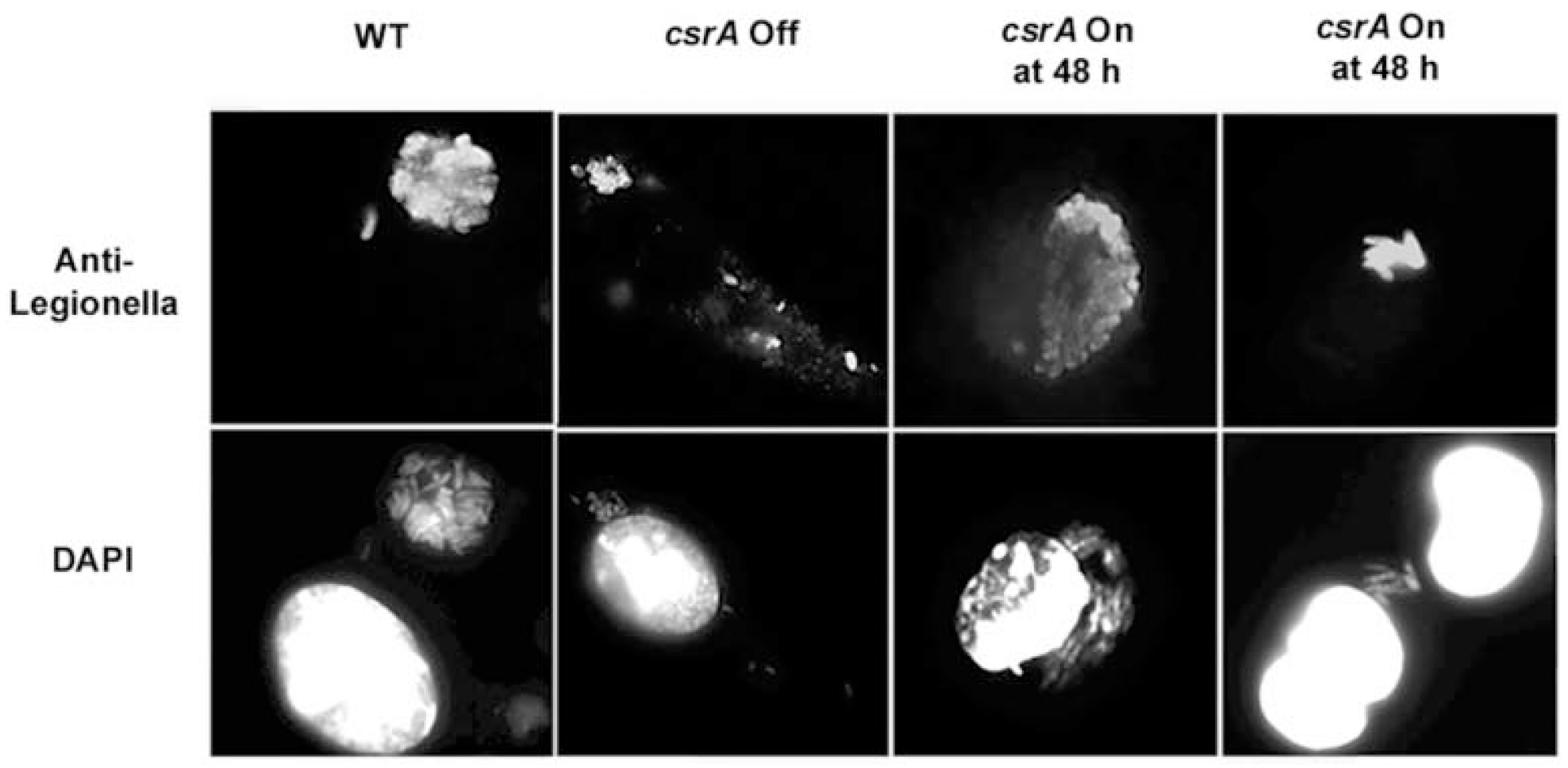
At 48 h after infection, *csrA::kan* mutants are intact and condensed. Macrophages were incubated for 48 h with wild-type Lp02 (MB110, column 1, ‘WT’) or E phase *csrA::kan* mutants (MB464, column 2, ‘*csrA* Off’), fixed, and double-labelled with anti-Legionella sera (top panels) and DAPI (lower panels) to visualize both intact and degraded bacteria and macrophage nuclei. Note the presence of a small replicative vacuole (column 2, top panel, upper left), degrading bacteria (column 2, top panel, centre), and an isolated microbe (column 2, top panel, bottom right), demonstrating the three intracellular phenotypes of *csrA* mutant bacteria observed in a single macrophage. In parallel, *csrA::kan* mutants were treated with IPTG 48 h post infection, incubated an additional 12–16 h, then prepared as described above (columns 3 and 4, ‘*csrA* On at 48 h’). Shown are representative images from three independent experiments performed in duplicate.

The fate of intracellular *csrA* mutants that persisted but did not replicate was examined in detail. Microscopy demonstrated that, by 48 h after infection, wild-type bacteria had replicated profusely (Fig. 6, column 1), whereas many of the *csrA* mutants persisted as single, intact rods (Fig. 6, column 2). In some macrophages, several either tightly packed or dispersed bacteria were seen, some of which were degraded. Those few *csrA* mutant bacteria that had replicated yielded 5–10 microbes per vacuole, appeared short and stubby, and stained poorly by DAPI (Fig. 6, column 2). To determine whether the *csrA* mutant cells that persisted in macrophages were viable, macrophages infected for 48 h were treated with IPTG, then incubated for an additional 12–16 h before microscopic analysis. Although a variety of phenotypes were again observed, many infected macrophages contained vacuoles of the replicative form of *L. pneumophila*, as evidenced both by their elongated form that stained brightly by DAPI or anti*Legionella* antibody and by the larger number of microbes per vacuole (Fig. 6, columns 3 and 4), neither of which was observed in the absence of IPTG treatment. Moreover, the *csrA* mutants that resided in macrophages for 48 h responded to IPTG treatment by replicating at approximately the same rate as wild-type *L. pneumophila*, as judged by the similar slope of the respective growth curves (Fig. 5B). Therefore, based on results of both microscopy and CFU assays, intracellular *L. pneumophila* must express *csrA* to differentiate to the replicative form.

## Discussion

This study demonstrates that *L. pneumophila* express the global repressor CsrA during the replication period to inhibit transmission traits and promote growth. When conditions deteriorate, LetA/S relieves CsrA repression to induce stress resistance and transmission to a new host. In particular, the phenotypes of *csrA* mutants and *csrA* constitutively expressing microbes indicate that, during the replication period, CsrA is an essential repressor of numerous traits characteristic of the PE phase, including pigment production, heat and osmotic resistance, and shape changes (Table 1; Supplementary material, Figs S2 and S3) and also the virulence-associated traits of motility, cytotoxicity, sodium sensitivity, and the ability to establish a vacuole protected from degradative lysosomes (Table 1; Figs 2–5). Thus, CsrA repression is vital during the replication period, but its activity must be alleviated by LetA/S to permit differentiation to the transmissive form.

Two lines of evidence indicate that CsrA repression is relieved by LetA to activate transmission phase traits. First, the phenotypic profile of *letA* mutants closely resembles that of microbes that constitutively express *csrA* (Figs 2–5; Supplementary material, Fig. S3; Table 1). Secondly, loss of CsrA bypassed the requirement for LetA as an inducer of every PE phase trait examined. Specifically, the *letA* mutant defects of cell shape, pigment production, stress resistance, motility, cytotoxicity, sodium sensitivity, and lysosomal evasion are a consequence of constitutive CsrA-mediated repression as they can be relieved by removal of CsrA (Table 1; Figs 2–4). Therefore, we propose that the major role of the activated LetA two-component response regulator is to counteract CsrA repression. Nevertheless, LetA/S may also regulate other as yet undefined traits independently of CsrA, as has been demonstrated in other bacterial species (Blumer et al., 1999; Suzuki et al., 2002).

The canonical Csr post-transcriptional regulatory system consists of both the protein CsrA and the non-coding inhibitory RNA CsrB. In other Gram-negative bacteria, LetA/S homologues induce expression of the CsrB regulatory RNA, which binds multiple copies of CsrA to relieve repression. Given that *L. pneumophila* encodes a LetA/S two-component system that antagonizes CsrA, it is also likely to utilize a CsrA-binding regulatory RNA. However, when CsrB and CsrC of *E. coli* or the functionally similar CsrB-like RNA species PrrB and RsmZ from *Pseudomonas fluorescens* (Aarons et al., 2000; Heeb et al., 2002; Weilbacher et al., 2003) were used as query sequences in a blastn homology search of the unfinished *Legionella* genome, no *L. pneumophila csrB* homologues were identified, consistent with previous results (Fettes et al., 2001). The primary sequence of *csrB* is not well conserved; instead, its secondary structure and CsrA protein binding sites are likely critical for its activity. Therefore, functional approaches will be needed to identify a putative *L. pneumophila* CsrB that is induced by LetA to counteract CsrA activity.

Even when CsrA repression is alleviated, *L. pneumophila* require the sigma factor FliA (sigma 28) to express the transmission traits of motility, infectivity, and cytotoxicity (Table 1; Fig. 4; data not shown). FliA is also necessary for *L. pneumophila* growth in certain amoebae (Heuner et al., 2002). Genetic data presented here and elsewhere indicate that *letA* mutation results in excess CsrA activity and loss of the RNA encoding the FliA sigma factor (Fettes et al., 2001). FliA is known to activate several class III flagellar genes involved in the terminal stages of flagellum development, including *flaA* (Heuner et al., 1997; Chilcott and Hughes, 2000). *Salmonella enterica fliA* mutants are defective not only for motility but also for macrophage cytotoxicity, epithelial cell invasion, and expression of components of the Type III secretory apparatus (Eichelberg and Galan, 2000). By analogy, *L. pneumophila fliA* mutants may lack cytotoxicity and infectivity due to failure to express not only flagella but also virulence effectors.

**Fig. 7.**
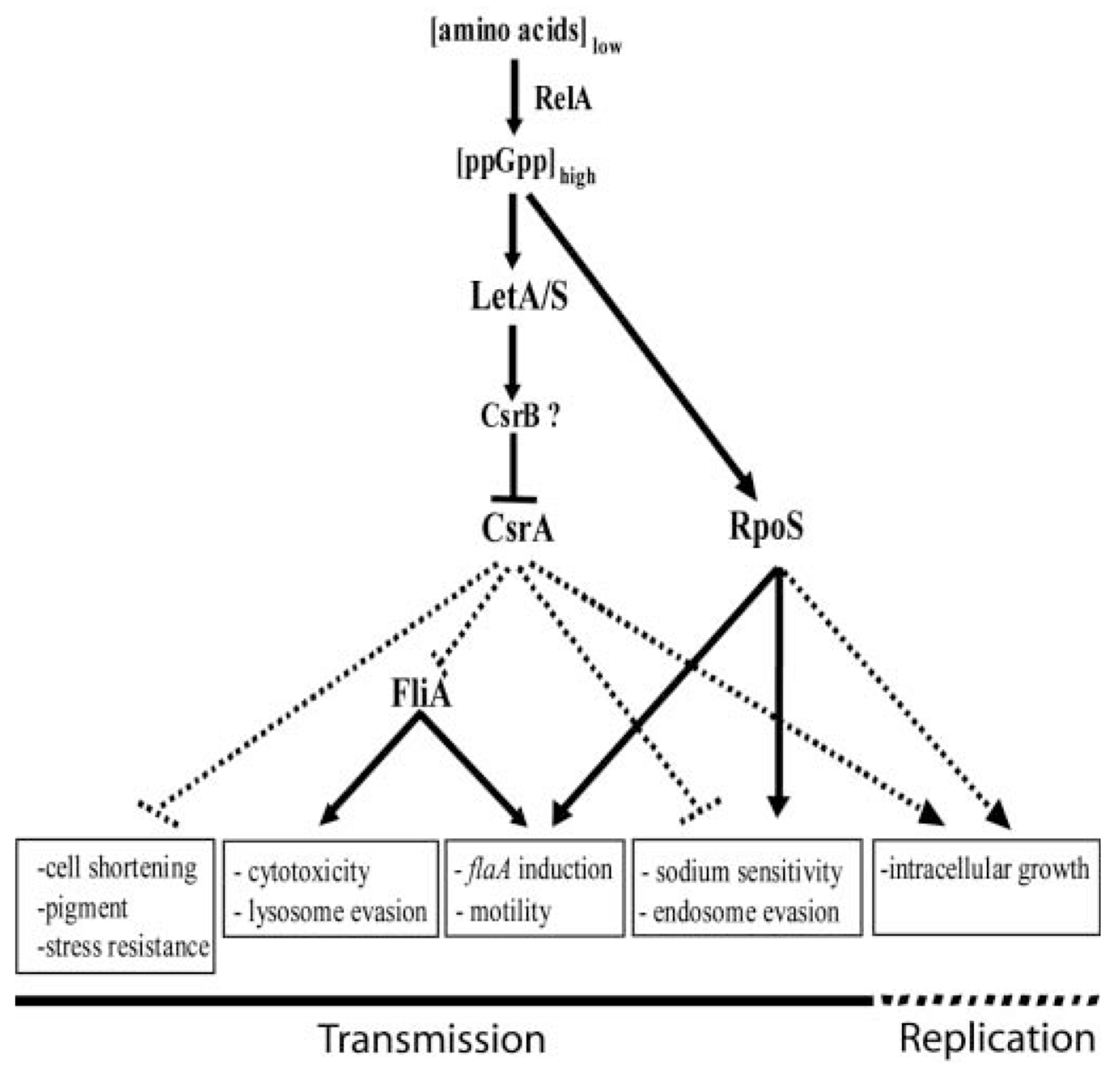
A model for regulation of *L. pneumophila* differentiation. Arrows indicate activation and bars indicate inhibition. Replication–phase regulatory interactions are represented by dashed lines, whereas PE phase regulatory pathways are indicated by solid lines. Transmission and replication phase phenotypes are labeled. CsrB has been predicted but not identified. For details, see text and other work (Hammer and Swanson, 1999; Bachman and Swanson, 2001; Hammer et al., 2002).

Based on the results herein, the model of *L. pneumophila* virulence regulation can be refined (Fig. 7). In response to elevated ppGpp, the two-component system LetA/S is activated and represses the activity of CsrA, likely by inducing the expression of an unidentified CsrB homologue. Relief of CsrA repression is sufficient to induce expression of PE phase traits such as cell shortening, pigment production, and heat and osmotic resistance. Additionally, loss of CsrA repression activates the expression of a number of virulence-associated traits, including cytotoxicity, motility, and evasion of phagosome-lysosome fusion, resulting at least in part from activation of the class II flagellar sigma factor FliA. CsrA may directly repress *fliA* mRNA stability or translation, or there may be an unidentified upstream activator of *fliA* expression that CsrA targets. In parallel, the stationary-phase sigma factor RpoS is also activated by ppGpp to induce a subset of PE phase traits independently of the LetA/S-CsrA pathway, including motility, sodium-sensitivity, intracellular growth and endosomal evasion (Bachman and Swanson, 2001).

*Legionella pneumophila* require CsrA to replicate efficiently in broth culture and in macrophages (Supplementary material, Fig. S1; Fig. 5). Similarly, *S. typhimurium csrA* mutants grow slowly in culture (Altier et al., 2000a). *Legionella pneumophila csrA* mutants’ premature expression of an array of virulence-associated traits may inhibit growth. However, two lines of evidence support the alternate hypothesis that CsrA is integral to *L. pneumophila* metabolism, independent of its repression of transmission factors. First, neither of two pleiotropic mutations, *fliA* and *dotA*, suppressed the growth defect of *csrA* mutants on solid or liquid media or in macrophages (data not shown). Second, attempts to recover by standard methods *csrA* null mutants in strains that lacked *letA, rpoS*, *fliA* or *letA rpoS* were unsuccessful, indicating that genetic inactivation of several transmission traits by any of these pleiotropic mutations is not sufficient to overcome the growth deficiency caused by a *csrA* mutation. Therefore, the poor growth of *csrA* mutants is not likely due to expression of virulence factors during the replication phase. Instead, by analogy to *E. coli csrA* or *uvrY (letA* homologue) mutants, we favour the hypothesis that *L. pneumophila csrA* mutants grow poorly due to imbalances in carbon flux and/or amino acid uptake (Wei et al., 2000; Pernestig et al., 2003). In *E. coli*, CsrA is a pro-glycolytic, antigluconeogenic global regulator, controlling numerous steps of carbon flux (reviewed in Romeo, 1998), and *L. pneumophila* is a fastidious bacterium that utilizes amino acids as its primary energy source (George et al., 1980; Hoffman, 1984). Perhaps the intracellular environment is especially stringent and prevents replication of *L. pneumophila csrA* mutants, whereas nutrient-rich broth culture supports slow replication of the microbes.

A remarkable finding is that even after extended incubations in macrophages, *csrA* mutants remain competent to replicate, provided *csrA* expression is first restored (Figs 5 and 6). In contrast, *dotA* conditional mutants induced to express *dotA* subsequent to macrophage infection do not replicate (Roy et al., 1998). Likewise, *csrA dotA* double mutants induced to express CsrA after macrophage infection failed to grow (data not shown). *Legionella pneumophila* must traffic immediately to an appropriate compartment to be competent to replicate (Roy et al., 1998). Whereas the Dot/Icm type IV secretion system is essential during phagocytosis but dispensable for replication (Coers et al., 1999), CsrA must be repressed for *L. pneumophila* to evade immediate phagosome-lysosome fusion, but must be active for intracellular replication that occurs in an acidic lysosomal compartment (Sturgill-Koszycki and Swanson, 2000).

While residing in macrophages for 48 h, *csrA* mutants acquire an atypical, condensed structure that stains poorly with DAPI and antibody (Fig. 6). After prolonged infection of HeLa epithelial cells, *L. pneumophila* differentiates into a spore-like Mature Intracellular Form (MIF) that is extraordinarily resilient and infectious (Faulkner and Garduno, 2002; Garduno et al., 2002). Because loss of *csrA* is predicted to lock *L. pneumophila* in the transmissive phase, their unusual morphology may indicate that during extended incubations in macrophages *csrA* mutants differentiate to MIFs.

Although in broth cultures a stringent response-like pathway is sufficient to activate numerous virulence traits of *L. pneumophila* (Hammer and Swanson, 1999), other signal transduction pathways are also likely to contribute to virulence expression. For example, even when CsrA activity was lacking in exponential phase cultures, *L. pneumophila* did not produce pigment nor become fully heat resistant or sodium-sensitive, indicating that these traits may also be regulated independently of CsrA. Furthermore, if the RelA alarmone acts solely via LetA/S to de-repress virulence traits, the *letA* and *relA* mutant phenotypes should be similar. *relA* mutants are partially defective for pigment production and motility, but unlike *letA* mutants, they are as sodium sensitive and cytotoxic as wild-type *L. pneumophila* (Hammer et al., 2002; Zusman et al., 2002). It is likely that *L. pneumophila* encodes redundant and overlapping mechanism(s) to regulate the transmissive phenotype, some of which may be independent of *relA* and the stringent-like response.

An unexpected result was the partial suppression of the sodium resistance of *dotA* mutants by a *csrA* mutation. The mechanism of sodium sensitivity is not understood, but the phenotype is linked to a functional type IV secretion system (Sadosky et al., 1993; Vogel et al., 1996) and the PE phase (Byrne and Swanson, 1998). However, not all sodium-sensitive microbes infect macrophages well (e.g. *fliA* mutants), nor do all sodium-resistant microbes fail to replicate (e.g. *letA* mutants), so the sodium sensitivity phenotype cannot be interpreted precisely. Perhaps loss of *csrA* restores partial function of the type IV secretory apparatus by activating compensatory genes in *lvh*, a region that encodes an alternate type IV secretion system that is dispensable for intracellular replication but contributes to conjugation (Segal et al., 1999). Interestingly, a *csrA* homologue *lvrC* maps within the *lvh* type IV secretion region (Segal et al., 1999), but its function is not known. Therefore, connections between CsrA, Icm/Dot, and Lvh may warrant further consideration.

Here we have demonstrated that CsrA functions at a regulatory crux, repressing transmission traits while activating replication of the intracellular pathogen *L. pneumophila*. Other pathogens may utilize a similar strategy to achieve reciprocal expression of two physiological states. In this model, intracellular pathogens are designed to either replicate efficiently or to promote their transmission to a new host, but not both. By doing so, microbes would minimize energy loss from production of extraneous transmission traits during the replication period and maximize rapid and coordinated differentiation to a transmissive form in response to environmental cues, thereby linking virulence intimately with general bacterial physiology.

## Experimental procedures

### Bacterial strains and culture

#### Legionella pneumophila

Lp02 (MB110), a virulent thymine auxotroph derived from the Philadelphia 1 strain (Berger and Isberg, 1993), was the parent for all the strains analysed (Supplementary material, Table S1). *Legionella pneumophila* was cultured in *N*-(2-acetamido)-2-aminoethanesulphonic acid (ACES, Sigma)-buffered yeast extract (AYE) broth at 37°C with agitation or on ACES-buffered charcoal yeast extract (CYE) agar at 37°C, both supplemented as necessary with 100 μg ml^−1^ thymidine (AYET, CYET). Bacteria obtained from colonies <2 weeks old were cultured in broth overnight, then subcultured in fresh AYET for an additional 16–24 h before experimentation. Exponential phase cultures (E) are defined as OD_600nm_ 0.3–2.0 and post-exponential cultures (PE) as OD_600nm_ 3.0–4.4. Where indicated, kanamycin (kan) was added to a final concentration of 25 μg ml^−1^, gentamicin (gent) to 10 μg ml^−^, chloramphenicol (cam) to 5 μg ml^−1^, and isopropyl-beta-D-thiogalactopyranoside (IPTG) to 200 μM (*in vitro* culture) or 1 mM (macrophage culture). To ascertain colony-forming units (CFU), serial dilutions of bacteria were incubated on CYET for 4 days and resultant colonies counted. All cloning was done in the *E. coli* DH5α strain using standard molecular techniques.

### Macrophage cultures

Bone marrow-derived macrophages were isolated from the femur exudates of A/J mice (Jackson Laboratory) and cultured as described (Supplementary material, Appendix A1; Swanson and Isberg, 1995).

### Phenotypic analysis of PE phase traits

Cytotoxicity of *L. pneumophila* for bone marrow-derived macrophages was quantified by incubating microbes in RPMI/FBS with macrophages for 1 h at various multiplicities of infection (MOI), then removing microbes and adding RPMI/FBS + 10% Alomar Blue (AccuMed) for 6–12 h (Supplementary material, Appendix A1; Byrne and Swanson, 1998; Hammer and Swanson, 1999). Sodium sensitivity was calculated by plating 10-fold serial dilutions of broth cultures into PBS onto CYET agar with or without 100 mM NaCl, then enumerating CFU after a 5–6 day incubation as described (Byrne and Swanson, 1998). Infectivity is a gauge of the ability of *L. pneumophila* strains to bind, enter, and survive inside murine bone marrow-derived macrophages during a 2-h incubation, as previously described (Supplementary material, Appendix A1; Byrne and Swanson, 1998). The ability of *L. pneumophila* strains to withstand a heat stress or osmotic shock was quantified essentially as described, with minor variations (Supplementary material, Appendix A1; Hammer and Swanson, 1999). Production of soluble pigment during late PE phase was measured spectrophotometrically (Wiater et al., 1994). At times shown, aliquots of broth cultures were centrifuged at 16 000 *g* for 10 min, then the OD_550nm_ of supernatants was determined. To assay bacterial density, bacterial pellets were resuspended to their original volume in PBS, and OD_600nm_ of a 1:10 dilution of the cell suspension was measured.

### *Promoter activity of* csrA *and* flaA *in broth cultures*

To assess *csrA* expression, the *csrA* promoter was fused to a promoterless *gfp* to generate the plasmid p*csrAgfp* (Supplementary material, Appendix A1), which was transferred to Lp02 by electroporation. To gauge promoter activity, GFP production by several independent clones of p*csrAgfp*, p*flaAgfp*, or pTPL6-*flaAgfp* containing *L. pneumophila* was quantified by fluorometry, as described (Supplementary material, Appendix A1; Hammer and Swanson, 1999).

### Fluorescence microscopy

The activity of the *csrA* and *flaA* promoters *in vivo* was quantified by fluorescence microscopy. Macrophages were infected at an MOI of <1.0 with PE phase cultures of Lp02 p*csrAgfp* (MB469) and Lp02 p*flaAgfp* (MB355). To synchronize the infection, microbes were centrifuged onto prechilled macrophage monolayers for 10 min at 400 *g*, and then incubated at 37° for an additional 10–30 min. Next, extracellular microbes were washed away and fresh media was added. At desired time points, coverslips were fixed for 30 min with prewarmed periodate-lysine-2.5% paraformaldehyde (McLean and Nakane, 1974), then washed extensively with PBS. Fixed cells were permeabilized with ice-cold methanol. Anti-*Legionella* rabbit serum (gift of Dr R. Isberg, Tufts University School of Medicine, Boston, MA, USA) was diluted 1:2000 into 2% goat serum in PBS and was detected by Texas Red-conjugated goat antirabbit secondary antibodies diluted 1:2000 (Molecular Probes). Incubations with antibody were done for 1 h at 37°C and were followed by several washes in 2% goat serum in PBS to reduce non-specific binding. Macrophage and bacterial DNA was stained with 4′,6-diamidine-2-phenylindole (DAPI) at 0.5 μg ml^−1^. Pilot experiments determined that 16 h post infection was optimal for visualizing vacuoles of replicating *L. pneumophila*, which were defined as tightly associated groups of five or more bacteria. Vacuoles were scored as GFP positive if >50% of the bacteria emitted green fluorescence above background. Duplicate coverslips were examined for each sample, and >50 replicative vacuoles were scored. Microscopy was performed with a Zeiss Axioplan 2 fluorescence microscope equipped with a 100× Plan-Neofluor objective of numerical aperture 1.3. Images were captured on a Spot digital camera (Diagnostics Instruments).

The percent of intact microbes after a 2 h incubation in macrophages was determined by fluorescence microscopy (Supplementary material, Appendix A1; Bachman and Swanson, 2001). *csrA* mutant strains and wild-type controls were also visualized by fluorescence microscopy after extended periods inside macrophages. At the desired time post infection, preparations were fixed and processed for fluorescence microscopy as described above for the promoter-gfp fusion experiments.

### *Construction of* csrA *conditional null alleles*

To create two *csrA* null alleles, first the *csrA* coding sequence was deleted and replaced with the kan or gent antibiotic resistance cassettes to create pGEM-DcsrA-Kan and pGEM-DcsrA-Gent (Supplementary material, Appendix A1). Next, the entire 4.2 kb or 3.6 kb mutant *csrA* genomic region from each plasmid was amplified by PCR using primers csrAup and csrAdown and the High Fidelity PCR kit (Roche), then the mutant allele was transferred by natural transformation (Stone and Abu Kwaik, 1999) into the wild-type Lp02 or the desired mutant background using the method of Dr Joseph Vogel (Washington University, St Louis, MO, USA), as described previously (Supplementary material, Appendix A1; Bachman and Swanson, 2001)

To transfer the *csrA* mutant allele onto the wild-type Lp02 chromosome, several independent natural competence experiments were performed with DNA amplified from pGEM-DcsrA-Kan and pGEM-DcsrA-Gent. Of ~50 independent antibiotic resistant colonies screened by colony PCR for the presence of a mutant-sized *csrA* locus, all colonies screened retained the wild-type sized *csrA* allele, whereas a control experiment performed in parallel yielded three of three desired homologous recombinants. When creation of a *csrA* mutant was attempted in *letA-22::kan* (MB414), *fliA-35::kan* (MB410), *rpoS120* (MB380), and *letA-22::kan rpos120* (MB461) mutant backgrounds, no *csrA* mutants were identified among the 5–10 colonies of each that were screened. Therefore, to bypass the apparent slow growth and/or lethality of *csrA* mutants, a conditional null strategy was adopted.

To generate a strain in which *csrA* expression could be induced, we recombined the mutant *csrA* chromosomal locus into a wild-type strain transformed with pMMB206Dmob-csrA (p206-csrA), a plasmid encoding a tightly regulated *csrA* ORF whose expression could be induced by IPTG (see Supplementary material, Appendix A1 for details).

Lp02 p206-*csrA* (MB477) was cultured with IPTG and 1 μg amplified DNA from either pGEM-ΔcsrA-Gent or pGEM-ΔcsrA-Kan, then transformants were selected on media that contained IPTG and the appropriate antibiotic. Several independent *csrA* conditional null clones containing either the gent (*csrA::gent* p206-csrA; MB465) or kan (*csrA::kan* p206-csrA; MB464) *csrA* alleles were isolated, verified by PCR and antibiotic resistance tests, then analysed in phenotypic assays. The conditional mutants were maintained on solid medium with appropriate antibiotics and 200 μM IPTG, as uninduced bacteria grew very poorly, and stable suppressors of the slow growth phenotype eventually arose after >5 days. When cultured in broth without IPTG, all clones displayed similar slow growth and premature motility and cytotoxicity during exponential phase, indicating that the phenotypes observed were due to the disrupted *csrA* locus and not unknown second site mutation(s). Conditional mutants maintained for 3–4 days in the exponential phase through serial backdilutions of broth cultures did not increase in growth rate, indicating suppressors of the slow growth phenotype did not readily arise in broth culture. To examine the effects of a *csrA* null phenotype in broth cultures, IPTG, which induces expression of *csrA* from the p206-csrA plasmid, was withdrawn as described elsewhere.

*csrA* double mutants were created by a similar strategy using the *csrA::gent* conditional null strain (MB465) or *csrA::kan* strain (MB460) cultured with IPTG as the recipient and *fliA, letA*, and *dotA* mutant PCR products (Supplementary material, Appendix A1) as the donor alleles. Several independent colonies of the putative *csrA* double mutants were verified by PCR to contain *fliA*, *letA*, or *dotA* mutant alleles, respectively, in combination with the *csrA* mutant locus. Appropriate antibiotic resistances were verified for all double mutants. Additionally, two independent isolates of each double mutant were assayed for broth growth, cytotoxicity and heat resistance; in all cases, the two isolates behaved similarly. These strains were maintained on CYET + IPTG to ensure expression of plasmid-borne *csrA*.

To study the phenotype of *csrA* single or double mutants, colonies were inoculated in AYET broth that contained IPTG/cam, cultured overnight to E phase, then washed and subcultured in AYET/cam broth without IPTG for ~16–24 h to allow intracellular stores of CsrA to deplete before experiments were performed. The wild-type control for all *csrA* mutant experiments was Lp02 p206-invcsrA (MB463). For infectivity, cytotoxicity, sodium-sensitivity, heat-resistance, and intracellular growth assays, CFU were plated on CYET/IPTG. As a test of genetic stability, dilutions were also plated on CYET/IPTG + cam, kan, or gent; no difference in CFU yield on medium ± antibiotics was noted, indicating stable maintenance of the *csrA*, *csrA fliA*, *csrA letA*, and *csrA dotA* alleles and the p206-csrA plasmid.

### CsrA constitutive expression plasmid

To complement *csrA* conditional null studies, a plasmid was engineered to yield constitutive expression of *csrA* when induced with IPTG. The ~1 kb *Nde*I-*EcoR*I fragment containing 350 bp 5’ and 400 bp 3’ to the *csrA* ORF obtained from pGEM-csrA was cloned into the MCS of pMMBGent-Dmob (Hammer and Swanson, 1999), a derivative of pMMB67EH (Frey et al., 1983). Similar to pMMB206, pMMBGent is an RSF1010 plasmid, but is marked with gent resistance and has a P_tac_ IPTG responsive promoter known to drive high levels of expression of the downstream gene (Hammer and Swanson, 1999). The resultant plasmid pMMBGentΔmob-csrA (pcsrA) was electroporated into wild-type Lp02 (MB472), *letA-22::kan* (MB476), *fliA-35::kan* (MB475), and *rpoS120* (MB474) (Hammer et al., 2002). Plasmid construction was verified by diagnostic restriction digest and complementation of an *E. coli csrA* mutant. pcsrA strains were cultured in AYET/gent + IPTG to maintain constitutive *csrA* expression. In all experiments with Lp02 pcsrA (MB472), wild-type Lp02 containing the empty pMMBGentΔmob vector (MB473) was used as a control strain.

## Supplementary Material

Fig S1 **Fig S1.** *L. pneumophila* requires the CsrA repressor to grow efficiently in broth.

Fig S2 **Fig S2.** LetA induces and CsrA represses coccoid cell morphology.

Fig S3 **Fig S3.** LetA/S activates and CsrA represses pigment production by PE phase *L. pneumophila*.

Table S1 **Table S1.** Bacterial strains, plasmids and primers.

Appendix **Appendix A1.** Supplementary experimental procedures.

The following material is available from http://www.blackwellpublishing.com/products/journals/suppmat/mmi/mmi3706/mmi3706sm.htm

## Figures and Tables

**Table 1.** Effects of CsrA on general PE phase phenotypes in *L. pneumophila.*

Strain	Growth phase	Motility^a^	Coccoid shape^b^	Pigment^c^	Heat-resistance^d^	Osmotic-Resistance^d^
Wild-type Lp02/vector (MB473)	E	−	+/−	−	− (0.08% ± 0.08%)	− (0.73% ± 0.37%)
Wild-type Lp02/vector (MB473)	PE	**++**	**++**	**++**	**++** (17% ± 7.8%)	**++** (24% ± 9.4%)
*csrA* (MB464, MB465)	E	**+**	**++**	−	**+** (9.4% ± 5.2%)	**ND**
*csrA* (MB464, MB465)	PE	**++**	**++**	**+++**	**++** (160% ± 60%)	**ND**
Lp02 pcsrA + IPTG (MB472)	PE	−	−	−	− (0.09% ± 0.08%)	− (0.50% ± 0.26%)
*letA*/vector (MB434)	PE	−	−	−	− (0.21% ± 0.16%)	− (0.94 ± 0.42%)
*csrA letA* (MB466)	PE	**++**	**++**	+**++**	**++** (66% ± 31%)	**ND**
*letA pcsrA* + IPTG (MB476)	PE	−	−	**+++**	**ND**	**ND**
*fliA/*vecto*r* (MB462)	PE	−	**++**	**+++**	**++** (14% ± 4.0%)	**++** (177% ± 148%)
*csrA fliA* (MB467)	PE	−	**++**	**+++**	**++** (32% ± 2.6%)	**ND**
*fliA pcsrA* + IPTG (MB475)	PE	−	−	−	− (0.15% ± 0.07%)	− (0.73% ± 0.13%)
*dotA* (MB460)	PE	**++**	**++**	**++**	**++** (106% ± 78%)	**ND**
*csrA dotA* (MB468)	PE	**++**	**++**	**++**	**++** (74% ± 27%)	**ND**
*rpoS/*vector (MB478)	PE	**+**	**++**	**++**	**++** (107% ± 50%)	**++** (67% ± 45%)
*rpoS pcsrA* +IPTG (MB474)	PE	−	−	−	− (0.73 ± 0.44%)	− (0.15% ± 0.06%)

a. Motility was gauged qualitatively by microscopy of wet-mounts and is based upon numerous independent observations. The motility of *csrA* mutants (MB464 465) increased as cultures progressed from early to late E phase. Wild-type Lp02 constitutively expressing *csrA* and carrying the pTLP6-*flaAgfp* reporter plasmid (MB470, +IPTG) expressed *flaA* at <30% the level of wild-type Lp02 pTLP6-*flaAgfp* (MB471), as measured by quantifying GFP fluorescence of PE phase bacteria by fluorometry.

b. PE phase *L. pneumophila* adopt a more coccoid, compact shape than E phase, replicating microbes. Shape was assessed qualitatively by microscopy of wet-mounts of numerous independent cultures. (++) indicates compact, wild-type PE shape (+/−) indicates longer, replicating shape, and (−) indicates extremely long shape (see Supplementary Fig. S2).

c. *L. pneumophila* secrete a melanin-like pigment in the PE phase. Representative graphs demonstrating pigment accumulation over time are shown in Supplementary Fig. S3. As actual absolute pigment values of wild-type strains varied between experiments and were affected by carriage of empty vectors, results are represented here as +++(exceeding wild-type pigment), ++(wild-type pigment levels), and – (very low pigment accumulation). For all experiments, a wild-type strain with appropriate empty vector was included as a reference. Data shown represent 2–6 independent experiments.

d. In the PE phase, *L. pneumophila* become resistant to a variety of environmental stresses, including heat and osmotic shock. (++) indicates >10% survival (+) indicates 1–10% survival, and (−) indicates <1% survival. Shown are the means of 2–3 independent experiments ± SEM. Data shown represent wild-type microbes carrying the empty pMMBGent-Dmob vector (MB473); similar results were obtained for wild-type strains carrying the pMMB206Dmob-invcsrA control vector (MB463).
