# Supplementary figures and images for: Legionella pneumophila CsrA is a pivotal repressor of transmission traits and activator of replication
Source: Mol Microbiol. Author manuscript; Available in PMC 2026 Jun 2. (PMC13227487; doi:10.1046/j.1365-2958.2003.03706.x)

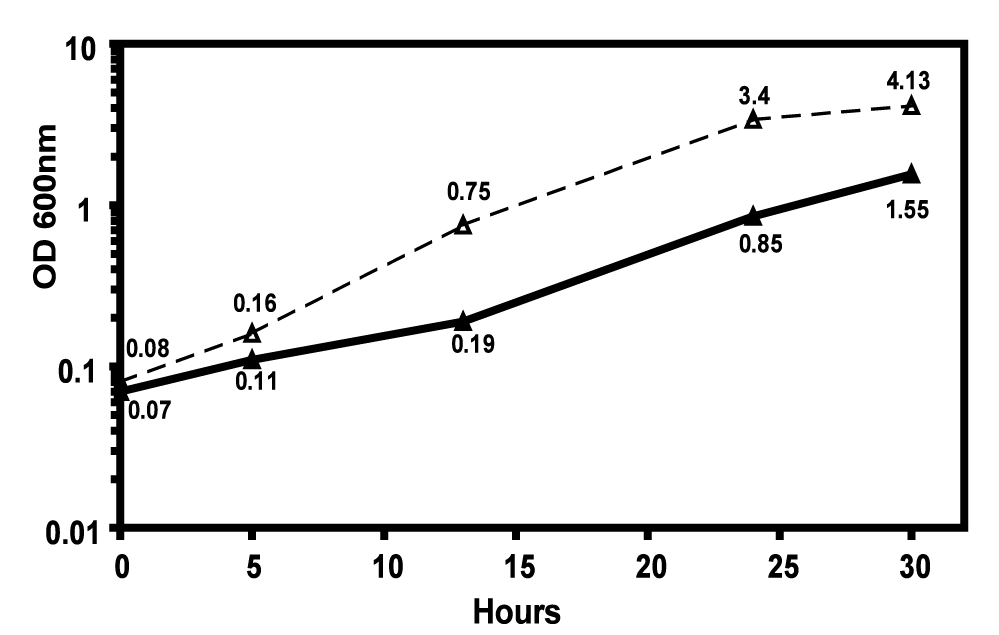

Supplement: Fig S1 — L. pneumophila requires the CsrA repressor to grow efficiently in broth. [file NIHMS2174200-supplement-Fig_S1.tif]

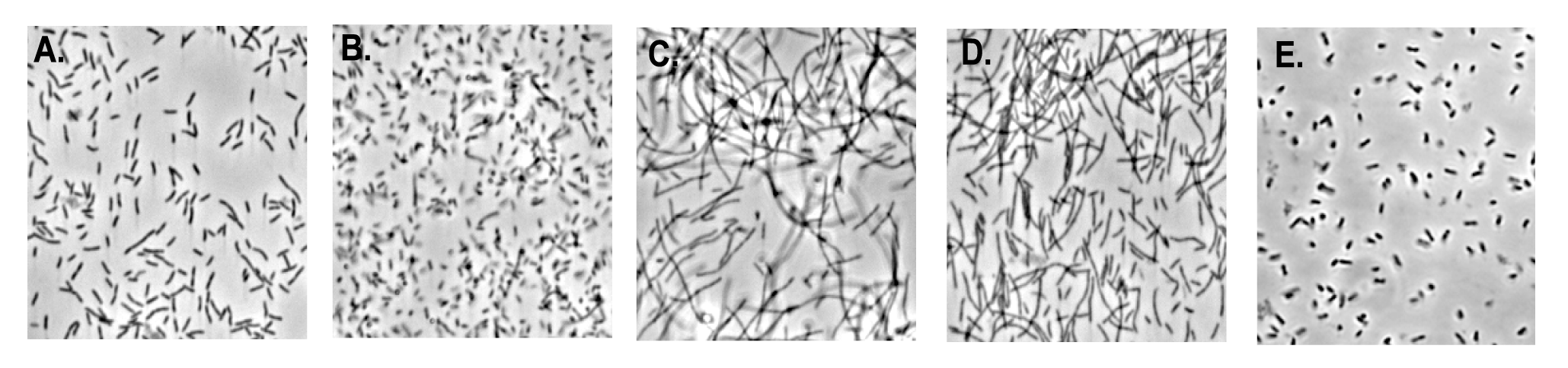

Supplement: Fig S2 — LetA induces and CsrA represses coccoid cell morphology. [file NIHMS2174200-supplement-Fig_S2.tif]

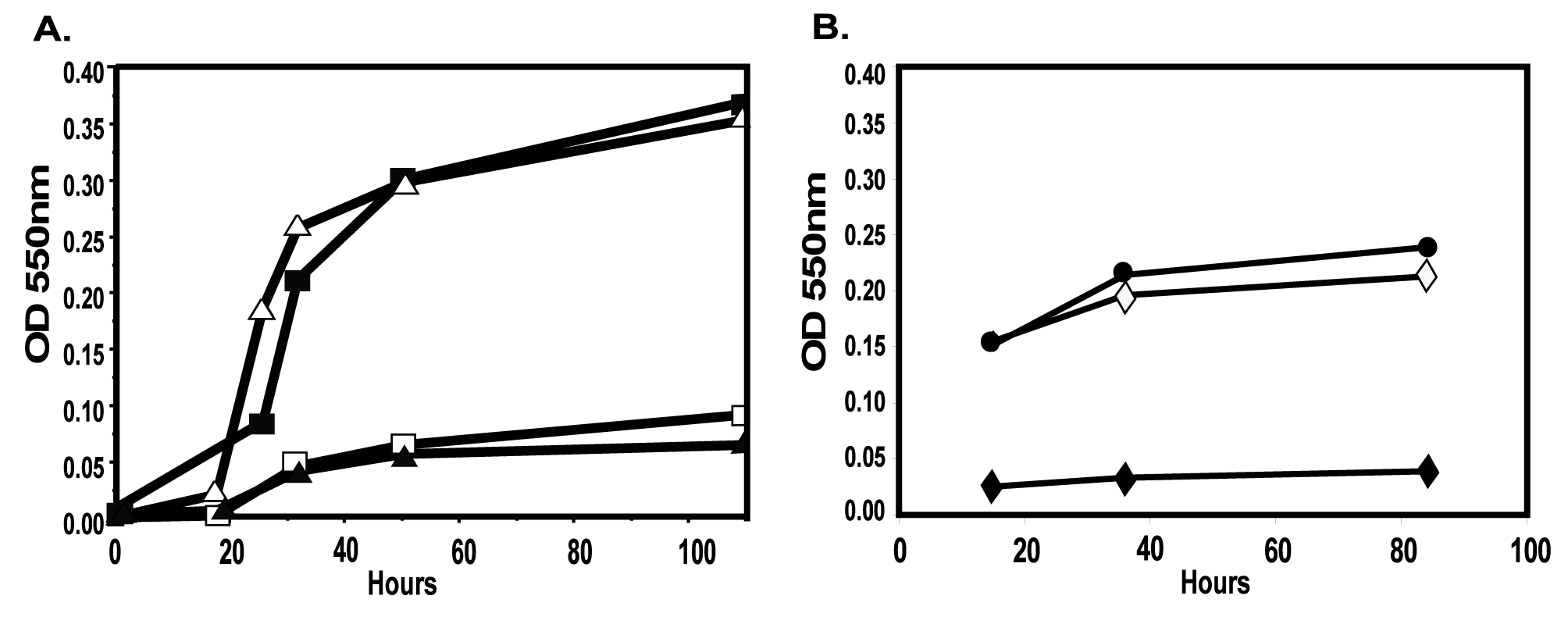

Supplement: Fig S3 — LetA/S activates and CsrA represses pigment production by PE phase L. pneumophila. [file NIHMS2174200-supplement-Fig_S3.tif]
