# Supplementary material for: Legionella pneumophila CsrA is a pivotal repressor of transmission traits and activator of replication
Source: Mol Microbiol. Author manuscript; Available in PMC 2026 Jun 2. (PMC13227487; doi:10.1046/j.1365-2958.2003.03706.x)
Supplement: Table S1 — Bacterial strains, plasmids and primers. [file NIHMS2174200-supplement-Table_S1.doc]

**­Supplemental Table 1. Bacterial strains, plasmids and primers**

Strain Relevant genotype/phenotype Reference

# E.coli

DH5α F-endA1 *hsdR17* (r- m+) *supE44* *thi-1* *recA1* *gyrA* (Nal^r^) Laboratory collection

*relA1* Δ(*lacZYA-argF*)_U169_Ф80dLacZΔM15*λpir*RK6

MG1655 Prototrophic glycogen accumulating parent strain for *csrA/csrB* mutants M. Cashel

TR5MG1655 MG1655 *csrA::kan*  Romeo *et al.* (1993)

RGMG1655 MG1655 *csrB::cam* Gudapaty *et al*. (2001)

MB326 DH5_ pUC4k, source of Kan^R^ Bachman and Swanson (2001)

MB500 DH5α pMMBGent-Δmob Hammer *et al*. (2002)

MB501 DH5α pGEM*-csrA*  This work

MB502 DH5α pGEM-*ΔcsrA-gent* This work

MB503 DH5α pGEM-*ΔcsrA-kan* This work

MB504 DH5α pcsrA This work

MB442 DH5α pMMB206-Δmob This work

MB505 DH5α p206-*csrA* This work

MB506 DH5α p206-*csrAinv*  This work

MB507 DH5α p*csrAgfp* This work

MB354 DH5α p*flaAgfp* Hammer and Swanson (1999)

*L. pneumophila*

MB110 Lp02 wild-type, Str^R^, Thy-, HsdR-

MB413 Lp02 *letA 22-3::kan* mutant Hammer *et al*. (2002)

MB416 Lp02 *letS 36::kan* mutant Hammer *et al*. (2002)

MB419 Lp02 *letE 121::kan* mutant Hammer *et al*. (2002)

MB410 Lp02 *fliA 35::kan* mutant Hammer *et al*. (2002)

MB460 Lp02 *dotA::gent* B. Byrne, unpublished

MB380 Lp02 *rpoS120* backcrossed EMS mutant Bachman and Swanson (2001)

MB461 Lp02 *letA 22-3::kan rpoS120* double mutant This work

MB434 Lp02 *letA 22-3::kan* pMMBGent-Δmob Hammer *et al*. (2002)

MB447 Lp02 *letE 121::kan* pMMBGent-Δmob Hammer *et al*. (2002)

MB462 Lp02 *fliA 35::kan* pMMBGent-Δmob This work

MB463 Lp02 p206-invcsrA 1A, vector control strain This work

MB464 Lp02 *csrA5::kan* mutant p206-*csrA* This work

MB465 Lp02 *csrA11:gent* mutant p206-*csrA* This work

MB466 Lp02 *csrA11:gent letA 22-3::kan* double mutant p206-csrA This work

MB467 Lp02 *csrA11::gent fliA 35::kan* double mutant p206-csrA This work

MB468 Lp02 *csrA5::kan dotA::gent* double mutant p206-csrA This work

MB469 Lp02 pcsrAgfp td(Δ)i This work

MB355 Lp02 pflAgfp td(Δ)I This work

MB470 Lp02 pTLP6-flaAGFP/ pcsrA This work

MB471 Lp02 pTLP6-flaAGFP/ pMMBGentΔmob, vector control strain This work

MB472 Lp02 p*csrA* This work

MB473 Lp02 pMMBGentΔmob, vector control strain Hammer and Swanson (1999)

MB474 Lp02 *rpoS120* p*csrA* This work

MB475 Lp02 *fliA 35::kan* p*csrA* This work

MB476 Lp02 *letA 22-3::kan* p*csrA* This work

MB477 Lp02 p206-*csrA* This work

MB478 Lp02 *rpoS120* pMMBGentΔmob This work

Plasmids

pMMBGent-Δmob pMMB67EH derivative, Δmob, lacI^q^, P_tac_, Gent^R^. Broad host range vector. Hammer and Swanson (1999)

Control for p*csrA* constitutive expression experiments.

pcsrA pMMBGent-Δmob with 1kb *csrA* genomic region ligated at MCS This work

lacI^q^, P_tac_, Gent^R^, inducible CsrA expression

pMMB206-Δmob pMMB66EH derivative, Δmob, lacI^q^,P_taclacUV5_, Cam^R^ Morales *et al.* (1993)

p206-*csrA* pMMB206-Δmob with 250bp Nco1/Cla1 fragment containing the *csrA* ORF This work

bluntly ligated at the BamHI site in the MCS, colinear with the

P_taclacUV5_ promoter, lacI^q^, Inducible CsrA expression

p206-*csrA*inv 1A pMMB206-Δmob with 250bp *csrA* ORF ligated in opposite direction This work

as P_taclacUV5_ promoter. Vector control for *csrA* mutant experiments

pUC4K source of 1.3kb kanamycin resistance GenBlock Pharmacia

pGEMT-Easy MCS within coding region of B-lactamase α-fragment Promega

linearized with single-T overhanges, Amp^R^

pGEM-*csrA* pGEMT-Easy with 2.6kb PCR amplified *csrA* chromosomal region ligated This work

into T overhangs, Amp^R^

pGem-*ΔcsrA-gent* pGem-*csrA* with 314bp BsrG1/Cla1 CsrA ORF fragment deleted, and a This work

1.9kb gentamicin cassete from pUC19-gent inserted at the HindIII site

90bp distal to deleted *csrA* ORF

pGem-*delcsrA-kan*  pGem-*csrA* with 314bp BsrG1/Cla1 *csrA* ORF fragment deleted, and a This work

1.3kb kanamycin cassette from pUC4k inserted at the HindIII site

90bp distal to deleted *csrA* ORF

p*flaA*gfp 150bp *flaA* promoter fragment fused to GFPmut3 in Hammer and Swanson (1999)

pKB5 with P_tac_ and lacI^q^ removed, td(Δ)i

p*csrA*gfp 450bp *csrA* promoter fragment fused to GFPmut3 in This work

pKB5 with P_tac_ and lacI^q^ removed, td(Δ)i

pTLP6-*flaA*gfp 150bp *flaA* promoter fragment fused to GFPmut3 in ColE1 replicon Hammer and Swanson (1999)

pTLP6, Cam^R^

Primers Sequence Wild-type Amplicon Size

csrApromoterup 5’-GGG AAT TCA ACA GAT AAT TTA AGG AAC AGA-3’

csrApromoterdown 5’-AGG GGA TCC AGC AGC TAC CTA CTT CTC C-3’ 450bp

csrAup 5’-TAT CTT TGG GGG CTT TGT CTA ATG -3”

csrAdown 5’-TCC ACC CTT GAT AAA CCT GAG TA -3’ 2.6kb

csrA1 5’-TGC AGG TAA AAT CCA GGC AAG- 3’

csrA2 5’-GGA CTG CTC CAA TAA CTT ACG TGA G- 3” 1.3kb

letA1 (gacA1) 5’-CGG CGG GGG AAT TTT ATC TT- 3’

letA2 (gacA2) 5’-GGA GGG CGG CTT CTT TTT ACT TC- 3’ 1.6kb

fliA1 5’-GAT GAA TAC AAA GAG ACG GAA GG- 3’

fliA2 5’-GAA GTC AAA GAT ACC CCC TAA GC- 3’ 7.5kb

dotAUpper2165L 5’-CGC ATT GGT ACT AGC CTT TCG TTA- 3’

dotALower2166L 5’-ACC ATC CTC ATA TTC CAC TTC CTT- 3’ 3.4kb
