# Supplementary material for: Legionella pneumophila CsrA is a pivotal repressor of transmission traits and activator of replication
Source: Mol Microbiol. Author manuscript; Available in PMC 2026 Jun 2. (PMC13227487; doi:10.1046/j.1365-2958.2003.03706.x)
Supplement: Appendix — A1. Supplementary experimental procedures. [file NIHMS2174200-supplement-Appendix.doc]

### Supplementary Experimental procedures

# Macrophage Cultures

Bone marrow-derived macrophages were isolated from the femur exudates of female A/J mice (Jackson Laboratory) as described ( Swanson and Isberg, 1995). After culture in L cell supernatant-conditioned media for 7 days, macrophages were gently removed from plates, collected by centrifugation, and resuspended in RPMI-1640 with 10% fetal bovine serum (RPMI/FBS; Gibco BRL). For microscopy and infectivity assays, macrophages were plated at 2-3 X 10^5^ cells per well in 24 well plates with or without circular coverslips (#1 thickness) respectively. For cytotoxicity assays, macrophages were plated at 5 X 10^4^ cells per well in 96 well plates.

*Cytotoxicity*

Cytotoxicity of *L. pneumophila* for bone marrow-derived macrophages was quantified by incubating microbes in RPMI/FBS with macrophages for 1 h at various multiplicities of infection (MOI), then removing microbes and adding RPMI/FBS + 10% Alomar Blue colorometric dye (AccuMed) for 6-12 h as described previously (Byrne and Swanson, 1998; Hammer and Swanson, 1999). Viable macrophages reduce the colorimetric dye, and the ratio of OD_570nm_ to OD_600nm_ for each well can be compared with a standard curve generated from assaying a range of viable macrophage concentrations, yielding the fraction of macrophages killed during the 1 h incubation. Absorbances were determined by a Spectramax 250 plate reader (Molecular Devices), and all samples were analyzed in duplicate or triplicate.

*Infectivity and intracellular growth*

Infectivity is a gauge of the ability of *L. pneumophila* strains to bind, enter, and survive inside murine bone marrow-derived macrophages during a 2 h incubation (Byrne and Swanson, 1998). Macrophages were incubated in RPMI/FBS + 100 μg ml^-1^ thymidine with *L. pneumophila* strains at an MOI of ~1 for 2 h, washed 3x with warm RPMI to remove extracellular microbes, then lysed in PBS by trituration. Serial dilutions of lysates were made in PBS and samples were plated on CYET +/- appropriate antibiotics. Infectivity was expressed as [(cell-associated CFU at 2 h)/ (CFU added at 0 h)] X 100. Intracellular growth assays followed the protocol described above, but after washing wells at 2 h post-infection, monolayers were incubated with 0.5 ml fresh RPMI/FBS supplemented with 100 μg ml^-1^ thymidine. Supernatants were subsequently collected at times indicated, and the remaining macrophages were lysed in PBS. CFU was calculated by plating serial dilutions in PBS of pooled supernatant plus lysate, yielding total bacteria per well. All infectivity and intracellular growth experiments were performed in duplicate wells for each strain at each time point. *csrA* mutant strains were incubated with macrophages in RPMI/FBS/thymidine +/- 1mM IPTG, and at 48 h post-infection, *csrA* mutants were induced +/- 1mM IPTG as indicated.

*Heat Resistance*

The ability of *L. pneumophila* strains to withstand a heat stress was quantified essentially as described (Hammer and Swanson, 1999) with minor variations. Cells from two parallel 0.75 ml aliquots of each broth culture were gently collected by centrifugation at 2300g for 5 min, then resuspended in fresh AYET. One aliquot was placed in a 57^0^ water bath for 20 min, while the control aliquot was placed in a 37^0^ water bath. Cultures were serially diluted in AYET, then CFU on CYET were enumerated. Heat resistance was calculated as [(heated sample CFU/ml)/ (control sample CFU/ml)] X 100.

*Osmotic shock resistance*

Resistance to osmotic shock was quantified generally as described (Hammer and Swanson, 1999). In brief, *L. pneumophila* from 0.75 ml of broth cultures were collected by centrifugation at 2300g for 5 min, then resuspended in AYET + 0.3 M KCl. AYET KCl cultures were next serially diluted in water (hypo-osmotic shock) or maintained in AYET KCL (control), then plated on CYET to enumerate surviving CFU. Control cultures treated only with water or AYET + KCl showed similar plating efficiencies, indicating no loss of CFU caused by either condition independently.

*Motility*

*L. pneumophila* motility was monitored qualitatively by examining wet-mounts of broth cultures by phase contrast microscopy using 40x or 100x objectives. Motility was defined as rapid, directed bacterial movement. To determine the growth phase of each sample, OD_600nm_ values of broth cultures were measured.

Construction of p*csrAgfp*

To create *pcsrAgfp*, a 450 bp region directly 5’ to the putative *csrA* ribosomal binding site was amplified from wild-type Lp02 chromosomal DNA by polymerase chain reaction (PCR) using the primers csrApromoterup and csrApromoterdown (Supp. Table 1). This region does not contain any apparent open reading frames or promoters for genes other than *csrA* (Columbia Genome Center Legionella Genome Project; http:///genome3.cpmc.columbia.edu/~legion/). The PCR product was ligated directly 5’ of the promoterless *gfp* gene to the *EcoR*I and *BamH*I sites of pBH6119, an RSF1010 plasmid that also encodes thymidylate synthetase as a selectable marker (Hammer and Swanson, 1999), resulting in p*csrAgfp*.

# Fluorometry

To gauge promoter activity, GFP production was quantified by fluorometry of p*csrAgfp*, p*flaAgfp*, and pTPL6-*flaAgfp* containing *L. pneumophila,* as described (Hammer and Swanson, 1999). To measure culture density, the OD_600nm_ of an aliquot of the appropriate broth culture was measured at each of the times indicated. Next, cells were collected by centrifugation, then diluted in PBS to an OD_600nm_ of 0.1. Relative fluorescence was quantified by a SPF-500C spectrophotometer (SLM Instruments) with an excitation of 488 nm, bandpass width of 2.5 nm, and emission of 510 nm, bandpass width 5 nm.

To monitor the effects of constitutive *csrA* expression on the promoter activity of *flaA*, the gene encoding the major component of the flagellum, the pTPL6-flaAgfp (Hammer and Swanson, 1999) plasmid was mated into the Lp02 pcsrA strain (*csrA* constitutive expression, MB472), creating MB470. pTPL6-*flaA*gfp is similar to the p*flaA*gfp and p*csrA*gfp reporter constructs described above, but is marked with Cam^R^ and has an origin of replication compatible with RSF1010 plasmids, including pcsrA.

*Fluorescent microscopy to determine percent intact microbes*

Infected macrophages were treated as described in infectivity experiments, but after washing with warm RPMI, coverslips were fixed and labeled with primary antibody and with a 1:2000 dilution of Oregon Green-goat anti rabbit secondary antibody. *L. pneumophila* was scored as intact if a distinct Oregon-Green positive rod shape was present. Non-intact bacteria were defined as particles of dispersed Oregon-Green positive fluorescence, or a rounded fluorescent vacuole, both indicative of degrading microbes (Fig. 4C).

# Natural Competence

Patches of *L. pneumophila* were cultured with 1μg DNA encoding the mutant allele for 2 days at 30^0^C, then transformants were selected on medium containing the appropriate antibiotic. The double recombination event to replace the wild-type gene was verified for several independent colonies by comparing the size of the product amplified from the candidates with that obtained from the corresponding wild-type Lp02 locus.

# Construction of csrA null plasmids

*L. pneumophila* *csrA* was identified by blastp searches against the Legionella database (Columbia Genome Center Legionella Genome Project; http:///genome3.cpmc.columbia.edu/~legion/)). A 2,664 bp genomic region surrounding the *csrA* open reading frame (ORF) was amplified by PCR from wild-type Lp02 colonies using the primers csrAup and csrAdown (Supp. Table 1), then this fragment was cloned into pGEMT-Easy (Promega), creating pGEM-csrA. Next, a 314 bp region containing the *csrA* ORF and a portion of the promoter was deleted by digestion with *BsrG*I and *Cla*I, then creating blunt ends which were religated, causing loss of both sites. A gent or kan resistance cassette was inserted at the *Hind*III site 90 bases distal to the former *Cla*I site. The 1.9 kb gent cassette was obtained by digesting plasmid pUC19-Gent with *EcoR*I, treated to generate blunt ends, and ligated to the *Hind*III site of the pGEM-ΔcsrA plasmid, creating pGEM-ΔcsrA-Gent. The kan cassette obtained from pUC4k as a 1.3 kb *EcoR*1 fragment was similarly treated, yielding pGEM-ΔcsrA-Kan. Resultant plasmids contained 1900 bp of genomic DNA 5’ to the deleted *csrA* coding sequence, the antibiotic resistance marker, and 350 bp 3’ to the deleted *csrA* coding sequence.

# Construction of p206-csrA

A 250 bp fragment containing the *csrA* ORF was obtained by digesting pGEM-csrA with *Nco*I and *Cla*I. After creating blunt ends, the fragment was ligated to the RSF1010 plasmid pMMB206 (kind gift of Dr. Eric Krukonis, University of Michigan, Ann Arbor, USA) after digesting with *BamH*I and Klenow to generate blunt ends. The resulting plasmid, p206-csrA, also harbors a deletion of a 400 bp *Age*I fragment that encodes *mobA*, as described previously (Segal and Shuman, 1998; Bachman and Swanson, 2001). Plasmid pMMB206 is derived from pMMB66EH, an RSF1010 derivative marked with Cam^R^, and has an IPTG responsive TacLacUV5 promoter with tightly controlled, low-level expression of sequences cloned downstream (Moral*es et a*l., 1991; Seifert, 1997; Lo*ng et a*l., 2001). As a control plasmid, we also retained pMMB206Δmob-invcsrA (p206-invcsrA), which contains the *csrA* ORF in the reverse orientation with respect to the TacLac promoter. After verifying that p206-csrA complemented the glycogen excess defect of the *E. coli csrA* mutant strain TR1-5MG1655 (a kind gift of Dr. T. Romeo, Emory University School of Medicine, Atlanta, GA, USA) and the control plasmid p206-invcsrA did not, the plasmids were transferred to wild-type Lp02 by electroporation, generating Lp02 p206-csrA (MB477) and Lp02 p206-invcsrA (MB463).

# Construction of csrA double mutants

To generate *csrA fliA* and *csrA letA* double mutants, the *fliA-35::kan* allele was amplified from MB410 using primers fliA1 and fliA2 and the *letA-22::kan* allele was obtained from MB413 using primers gacA1 and gacA2; the *dotA::gent* allele was amplified from MB460 with primers dotAUpper2165L and dotALower2166L (B. Byrne and M. Swanson, unpublished). All mutant PCR products were utilized in natural competence procedures, as described above.
